# Immune-related gene risk model establishment and role of key gene FUCA1 in malignant pleural mesothelioma

**DOI:** 10.3389/fphar.2025.1577232

**Published:** 2025-05-23

**Authors:** Lin Shi, Dongqi Yuan, Fuyi Zhu, Yuchao He, Ran Zuo, Liwei Chen, Yi Luo, Yu Wang, Dingzhi Huang, Peng Chen, Hua Guo

**Affiliations:** ^1^ Department of Tumor Cell Biology, Tianjin Medical University Cancer Institute and Hospital, Tianjin, China; ^2^ Department of Thoracic Oncology, Lung Cancer Diagnosis and Treatment Center, Tianjin Medical University Cancer Institute and Hospital, Tianjin, China; ^3^ National Clinical Research Center for Cancer, State Key Laboratory of Druggability Evaluation and Systematic Translational Medicine, Tianjin’s Clinical Research Center for Cancer, Key Laboratory of Cancer Prevention and Therapy, Tianjin, China; ^4^ Department of Oncology, Inner Mongolia Autonomous Region People’s Hospital, Hohhot, China; ^5^ Beijing Children’s Hospital, Capital Medical University, Beijing, China; ^6^ Department of Integrated Chinese and Western Medicine, Tianjin Medical University Cancer Institute and Hospital, Tianjin, China

**Keywords:** malignant pleural mesothelioma, immune cell, machine learning, FUCA1, EMT

## Abstract

**Background:**

Malignant pleural mesothelioma (MPM) is a rare type of tumor closely associated with asbestos exposure. Increasing evidence shows that high immuno-heterogeneity reduces the therapeutic efficacy of MPM. At present, good biomarkers to screen immunodominant populations and predict the efficacy of immunotherapy are lacking.

**Methods:**

In this study, expression data from TCGA, GSE2459, GSE51024, and GSE29354 were integrated for model construction. An eight-gene risk score model (FLI1, IL32, FUCA1, CCR2, PSMB10, CCL5, WT1, and KRT5) was constructed using CIBERSORT, weighted gene co-expression network analysis, Cox regression analysis, differentially expressed gene analysis, and protein–protein interaction network. The K–M survival analysis was used to evaluate the prediction ability of the risk score model. The TIDE database and Oncology Drug Sensitivity Genomics database were used to assess the predictive power of risk score models for treatment. In addition, the expression of the key gene in para-carcinoma tissue and MPM samples were detected by Immunohistochemistry. Patient clinical information was employed to evaluate the relationship between key genes and patient survival. Finally, the biological functions of the key gene were examined by in vitro and in vivo experiments.

**Results:**

The score model was used to divide patients with MPM into low- and high-risk groups. The high-risk group was characterized by a survival disadvantage, and they were less sensitive to immunotherapy. Clinical data suggest that FUCA1, which is a key gene in the model, is an independent risk factor for predicting the prognosis of patients with MPM. A series of experiments demonstrated that FUCA1 expression was negatively correlated with the proliferation, invasion and migration abilities of MPM cells. Further studies revealed that FUCA1 inhibited epithelial–mesenchymal transition in MPM cells by regulating the PI3K-AKT signaling pathway.

**Conclusion:**

The risk score model provides a new perspective for screening potential populations to benefit from immunotherapy and predicting their survival. FUCA1 may be a potential prognostic biomarker and promising therapeutic target for patients with MPM.

## 1 Introduction

Malignant pleural mesothelioma (MPM) is a rare tumor of high invasiveness deriving from pleura mesothelial cells, the mechanism of which is closely related with asbestos exposure (Broeckx and Pauwels, 2018). Intake of asbestos fibers causes cytokine production, which leads to reactive oxygen species from the topical area of inflammation that induces carcinogenesis (Betti et al., 2018). With the increasing consumption of asbestos in developing countries, MPM has become a sustainable global health problem (Tsao et al., 2009; Cinausero et al., 2019). The onset of MPM is insidious, with a latency period of about 40 years. Most patients are usually diagnosed at an advanced stage, which is difficult to be surgically resected, with high mortality. Despite advancements in treatment, the prognosis for patients with MPM remains poor, and therapeutic efficacy is often limited (Ahmadzada et al., 2018). Although the correlation between MPM and molecular pathways has already been determined, such as cell cycle regulation, cell apoptosis, growth factors, and angiogenesis (Bronte et al., 2016), targeted therapies for MPM have not yet been discovered.

MPM is divided into three subtypes in accordance with cellular morphology: epithelioid, sarcomatoid, and biphasic (mixed); among them, the prognosis and response of chemotherapy for epithelioid are the best, whereas those for sarcomatoid are the poorest (Verma et al., 2018). The outcomes of the Check Mate-743 clinical trial facilitated the recommendation of double immune checkpoint inhibitors (ICIs) as a first-line treatment for MPM (Baas et al., 2021). Clinical trials have demonstrated the efficacy of dual immunotherapy with navulizumab and ipilimumab in patients with nonepithelial pleural mesothelioma. However, the problem of high inter-individual variability in efficacy still exists. Commonly used predictive parameters for immunotherapeutic efficacy are PD-L1 expression (Powles et al., 2014), tumor mutational burden (TMB) (Snyder et al., 2014), microsatellite instability (MSI) (Dudley et al., 2016), and CD8 T cells (Ghatalia and Plimack, 2019). However, in patients with MPM, these predictive parameters do not have a good predictive power. Even in layers with high PD-L1 expression, the benefit of immunotherapy has not yet been seen for the whole population (Rijavec et al., 2022). Hence, obtaining new biomarkers to screen populations that may be effective for ICI treatment is critical.

The tumor microenvironment (TME) has an important role in the pathogenesis and therapeutic effect of MPM. In recent years, studies have recognized it as an important complement for predicting relapse and mortality for TNM staging systems by evaluating the enrichment of tumor-infiltrating lymphocytes (Mlecnik et al., 2011; Pagès et al., 2018). Traditional, flow cytometry, and immunohistochemical staining assays for tumor immune infiltration are incapable of evaluating the effect of immunity all-around due to the quantity limitation of immune markers. Therefore, the constant accumulation of transcription data has provided a reliable resource for large-scale immune spectacle analysis (Finotello and Trajanoski, 2018).

In this study, an eight-gene risk score model was constructed on the basis of MPM immune microenvironment characteristics. The risk score model was able to well predict the prognosis of patients with MPM and the efficacy of immunotherapy and chemotherapy. An in-depth analysis of the key genes in the risk score model was conducted. The results showed that FUCA1 could inhibit the proliferation, invasion, and migration of MPM cells, and that it may play a tumor-suppressive role by inhibiting epithelial–mesenchymal transition (EMT) through the PI3K-AKT signaling pathway. These findings provide novel insights into personalized and precise treatment strategies.

## 2 Materials and methods

### 2.1 Data collection

The gene expression profiles and clinical features of MPM samples were derived from the database combination of The Cancer Genome Atlas (TCGA, http://tcga-data.nci.nih.gov/tcga/) and Gene Expression Omnibus (GEO, http://www.nc-bi.nlm.nih.gov/geo). A total of 289 samples were enrolled in this study [GSE2459 (N = 54), GSE51024 (N = 96), GSE29354 (N = 53), and TCGA (N = 86), Supplementary Table S1]. The batch effects among different datasets were removed using the “ComBat” method in the SVA R package (Wu et al., 2020). Moreover, duplicate samples and cases without clinical outcomes in the downloaded data were removed. Counts data were processed as TPM format with Toil. Pan-cancer gene expression data were extracted from TCGA for further validation. The validity of immune risk markers was verified by treating the advanced urothelial carcinoma cohort (IMvigor210 cohort) with atezolizumab (anti-PD-L1 mAb) immunotherapy (Mariathasan et al., 2018). The detailed clinical comments and complete gene expression profiling for the anti-PD-L1 cohort can be found in IMvigor210CoreBiologies (http://researchpub.gene.com/). The response of patients with MPM to immunotherapy and the T-cell functional status were indirectly predicted by the non–small cell lung cancer (NSCLC) immunotherapy cohort in the Tumor Immune Dysfunction and Exclusion Database (TIDE) (http://tide.dfci.harvard.edu/).

### 2.2 CIBERSORT analysis

The ratios for 22 types of tumor-infiltrating immune cells (TIICs) in each sample were calculated with “CIBERSORT” in the R package. The relative expression levels of 11,758 genes in individual tissue samples were analyzed on the basis of gene expression profiling using CIBERSORT(Newman et al., 2015), and the ratios for 22 types of TIICs in each tissue were predicted and transformed from the standard MPM gene expression profiling. The relative expression levels of 22 TIICs in each sample were tested. Further analyses were performed in accordance with the results of statistical significance (*p* < 0.05).

### 2.3 Weighted co-expression network analysis (WGCNA)

WGCNA was used to describe the gene association patterns among different samples and recognize the highly covariant gene sets, candidate biological mark genes, or therapeutic targets in accordance with the relativity of each gene set and the association between gene sets and phenotypes. A co-expression network of 11,758 genes was constructed using WGCNA in the R package. The scale-free topology guaranteed the soft threshold power value b to be 9. Modules were defined by adopting the layered clustering method with weighted coefficient matrices. Genes related to immune cells were obtained by obtaining gene modules related to immune cells in MPM. Analyses were performed following the instruction of packages (Langfelder and Horvath, 2008).

### 2.4 Survival analysis and subtyping

Genes in the group module that were most associated with immune cells were selected. Univariate Cox regression and Kaplan–Meier (K–M) analysis were performed using “Survival” and “Survminer” in the R package. Datasets of immune cell-related genes associated with the OS of patients were identified. Five immune cell-related genes of independent prognostic significance were defined with the multivariate Cox regression analysis, and a reliable patient cluster was recognized using “ConsensusClusterPlus” in the R package, the maximum of clustering being 7. ClusterAlg = “pam”, innerLinkage = “Pearson”. A clustering heatmap was constructed using “pheatmap” in the R package.

### 2.5 Immune infiltration analysis for different subgroups

The composite abundance of immune cells in samples was predicted using five algorithms EPIC, MCPcounter, xcell, ssGSEA, and quantiseq of “Immunedeconv” in the R package. The expression levels of PD-L1, CTLA-4, LAG-3, and other immunosuppressive factors were compared. Tumor purity and stromal and immune cell analysis were carried out using “Estimate” in the R package.

### 2.6 Mutational analyses

Clinical information of MPM mutational data and samples was downloaded from the TCGA database. Mutational data were analyzed and visualized using “maftools” in the R package to identify the somatic mutations of patients with MPM.

### 2.7 Evaluation of MSI

“PreMSIm” is an R package for predicting MSI in accordance with the data of transcriptome, and it was used to predict the microsatellite instability status of 289 patients with MPM. The results were classified as MSI high and MSI low.

### 2.8 Identification of differentially expressed genes (DEGs)

The data of gene expression profiling for each patient were standardized using “limma” in the R package. The empirical Bayes method in the “limma” package was adopted to determine DEGs for patients in the latter two clusters after consensus clustering to identify key factors associated with prognosis and immune cell infiltration. DEGs with significant differences were selected with *p* < 0.05 and |logFC| ≥ 1 as the criteria.

### 2.9 Functional annotation and gene set enrichment analysis (GSEA)

Gene Ontology (GO) enrichment analysis was performed using “ClusterProfiler” in the R package to explore the potential biological processes related with the acquired DEGs (Ritchie et al., 2015). Enrichment analysis was conducted for GO from three aspects: biological process (BP), molecular function (MF), and cellular component (CC). The biological pathways of activation or inactivation in patients of the latter two clusters were identified after consensus analysis by performing GSEA on the expression data after regulation of all transcriptomes.

### 2.10 Protein–protein interaction (PPI) network establishment and hub gene identification

A PPI network was established by adopting the Search Tool for the Retrieval of Interacting Genes (STRING) database, and the relationship between DEGs of patients in the latter two clusters was determined after consensus clustering. In the STRING database, the interaction score was set as 0.4. Interaction data were obtained in the STRING database, and the PPI network was more readable through Cytoscape. Central hub genes that were directly interacted with DEGs and subordinate hub genes that directly interacted with central hub genes were identified.

### 2.11 Generation of prognostic indicators based on key molecules

The optimal prognostic risk model with genes obtained after performing univariate COX and multivariate COX analysis and hub genes of DEGs after performing consensus analysis was constructed using the “survival” R package to identify immune cell related genes associated with survival. The risk scores were calculated for each patient by using the following formula: Riskscore = coef (FLI1) × expr (FLI1) + coef (IL32) × expr (IL32) + coef (CCR2) × expr (CCR2) + coef (FUCA1) × expr (FUCA1) + coef (PSMB10) × expr (PSMB10) + coef (CCL5) × expr (CCL5) + coef (WT1) × expr (WT1) + coef (KRT5) × expr (KRT5).

In accordance with the median value of risk scoring, patients with MPM were divided into high-risk group and low-risk group. K–M survival analysis was conducted using “survival” and “survminer” in the R package to evaluate the survival difference between the two groups.

### 2.12 Construction of a nomogram in accordance with risk score and clinical traits

The MPM datasets were divided into testing dataset (50%) and validation (50%) dataset to evaluate the predictive power of risk scoring. The survival difference between the two patient groups was evaluated with K–M survival analysis using “survival” and “survminer” in the R package. Clinical features, including BAP1, stage, gender, age, and risk score, were included in the multivariate Cox analysis for establishment of the predictive model. Finally, a nomogram that included risk score and clinical pathological features was constructed using “rms” in the R package.

### 2.13 Prediction of response to immunotherapy and chemotherapy

By adopting the NSCLC immunotherapy cohort in the TIDE database, response to immunotherapy and T-cell function and state in patients with NSCLC were predicted. The imported data were standardized in accordance with the requirements of TIDE database. The default cutoff value of TIDE scoring was 0. The efficacy of the risk score model was indirectly validated in the atezolizumab-treated advanced uroepithelial cancer immunotherapy cohort (IMvigor210 cohort). The prediction of response to chemotherapy for each sample was based on the Oncology Drug Sensitivity Genomics (https://www.cancerrxgene.org/) predicted by the largest public pharmacogenomics database. Four commonly used chemotherapy drugs for MPM are cisplatin, etoposide, gemcitabine, and vinorelbine. Prediction was implemented using “pRRophetic” in the R package. The half-maximum inhibitory concentration (IC50) of the sample was evaluated in accordance with the previous description.

### 2.14 Patients and tissue specimens

A retrospective study was conducted on the basis of formalin-fixed paraffin-embedded tumor samples of 34 patients with MPM from Tianjin Medical University Cancer Institute and Hospital (Tianjin, China) between January 2013 and July 2021. The study conformed to the ethical guidelines of the Helsinki Declaration, and it was approved by the Ethics Committee (No. bc2022241).

### 2.15 Immunohistochemistry

MPM tissue sections were dewaxed in xylene and rehydrated in ethanol series. Antigen was extracted in citrate or EDTA, which was then treated with 3% hydroxides for 10 min to inhibit the activity of endogenous hydrogen peroxide. Then, the samples were stained for 30 min with antibodies at room temperature and kept overnight at 4°C. After being washed, tissue microarrays and sections were incubated with secondary antibodies for 1 h at room temperature, observed with 3,3-diaminobenzidine solution (ZSGB-Bio), and counterstained with hematoxylin. The percentage of immunoreactivity scoring was classified on a four-point scale: 0 was assigned to <10% positive cells; one was assigned to 10%–40% positive cells; two was assigned to 40%–70% positive cells; and three was assigned to 70%–100% positive cells.

### 2.16 Cell culture

BEAS-2B cells were purchased from American Type Culture Collection. NCI-H2452 and NCI-H2052 cells were purchased from the Cell Bank of Chinese Academy of Sciences (Shanghai, China). In accordance with culture requirements (37°C, 5% CO_2_), cells were cultivated in 1,640 medium (HyClone, Logan, UT, USA) and supplemented with 10% fetal bovine serum (FBS; Zetalife, USA) and 1% penicillin/streptomycin (PS; HyClone, Logan, UT, United States).

### 2.17 Transfection assay

Transfection assay was performed to obtain lentiviral particles. Packaging plasmids (VSVG and PAX2) and expression plasmids (sh-FUCA1, sh-Ctrl, FUCA1, and Vector) were transfected into HEK293T cells by using PEI (Polysciences, Warrington, USA). Lentiviruses were produced by HEK293T cells. NCI-H2452 and NCI-H2052 cells were infected with a lentivirus to produce stable FUCA1 overexpression and knockdown cells under puromycin (Gibco, New York, United States) selection.

### 2.18 Western blotting (WB) analysis

Proteins were extracted from cells, which were lysed with 1 × SDS lysis buffer (Tris–HCl, pH 6.8, 62.5 mM, 2% SDS, 10% glycerol) supplemented with 1 mM NaF, 1 mM Na_3_VO_4_, 1 × protease, and phosphatase inhibitor cocktail (Hoffman-la Roche Ltd., Basel, Switzerland) on ice for 30 min. Then, the proteins were separated by SDS-PAGE and transferred to a PVDF membrane (Merck Millipore). Next, they were blocked with bovine serum albumin (BSA), followed by incubation with primary and secondary antibodies. The information of primary antibodies is as follows: FUCA1 antibody (1:1000, Proteintech), E-Cadherin antibody (1:1000, BD Pharmingen), Vimentin antibody (1:1000, Abcam), Snail antibody (1:1000, Santa Cruz), PI3K antibody (1:1000, Proteintech), p-PI3K antibody (1:1000, Abmart), AKT antibody (1:1000, Cell Signaling Technology), p-AKT antibody (1:1000, Cell Signaling Technology), and GAPDH antibody (1:1000, Cell Signaling Technology).

### 2.19 Quantitative real-time PCR

Total RNA was collected and homogenized in TRIzol (Ambion, USA). RNA reverse transcription and extraction were carried out following standard procedures. cDNA was synthesized by reverse transcription of the isolated RNA based on a nine kit (Takara, Japan). Amplification reaction was conducted using predesigned primers in accordance with the manufacturer’s instructions (Takara, Japan). The results were normalized with GAPDH. The primers involved in the experiment are detailed in Supplementary Table S2.

### 2.20 CCK-8 assay

Cells were seeded in 96-well plates at 1,000 cells/well and cultured for 24, 48, 72, and 96 h. Then, CCK-8 solution (Biosharp, Beijing, China) was added to each well for one or two h at 37°C, and the absorbance was measured at 450 nm by using a microplate reader (Molecular Devices).

### 2.21 Colony formation assay

Cells were seeded in six-well plates at a density of 1,000 cells per well and cultured at 37°C for 2 weeks. Subsequently, the plates were washed with phosphate-buffered saline (PBS) and fixed with 4% paraformaldehyde for 15 min. Finally, 0.1% crystal violet was used to stain the plates. The colonies were counted with ImageJ software (Wayne Rasband, National Institutes of Health, United States).

### 2.22 EdU proliferation assay

Cells were seeded in 12-well plates. EdU assay was conducted for cell proliferation detection using the BeyoClick EdU Cell Proliferation Kit (Beyotime, Shanghai, China) with Alexa Fluor 594. The cell nucleus was detected with DAPI staining solution. After the cells were washed in PBS, they were studied using an inverted microscope.

### 2.23 Transwell assay

Cells were seeded in the upper chamber at a density of 1 × 10^5^ cells per well. The lower chamber was filled with 600 µL of RPMI 1640 medium containing 20% FBS. After being incubated for 12 h, the cells were fixed and stained with crystal violet. The cells in the upper chamber were removed, and the migrated cells were photographed and counted with ImageJ software.

### 2.24 Wound healing assay

Cells were seeded in six-well plates, and monolayers were scratched with a 10 µL pipette tip until 95% confluence. The cells were subsequently photographed every 3 h, and the migrated areas were calculated using ImageJ software.

### 2.25 Immunofluorescence

Cells were seeded in 12-well plates, fixed in 4% paraformaldehyde (Cat# P0099, Beyotime Biotech. Inc.), permeabilized in 0.2% Triton X-100, and blocked with 5% BSA. Immunofluorescence staining was performed using anti-FUCA1 and anti-Vimentin antibody. The secondary antibodies used were Alexa Fluor 488 and 594 anti-mouse IgG, and they were incubated with 1 μg/mL DAPI. Imaging was carried out using a microscope.

### 2.26 Xenograft models

The animal experiments were approved by the Ethics Committee of Tianjin Medical University Cancer Institute and Hospital (No. 2023054), Tianjin, China. Female BALB/c nude mice (4–5 weeks old) were purchased from SPF Biotechnology (Beijing, China) for xenograft animal assays. For tumor growth assays, the mice were randomly divided into two groups (FUCA1 vector and FUCA1 OE; n = 5 per group). The prepared NCI-H2052 FUCA1-Vector/NCI-H2052 FUCA1-OE tumor cells (5 × 10^6^) in 100 μL PBS were injected subcutaneously. Tumor volumes were measured every 5 days from 5 days after the injection. The mice were humanely sacrificed on day 40. Tumor growth was analyzed by measuring tumor length (a) and width (b) in accordance with the following formula: volume (mm^3^) = ab^2^/2.

### 2.27 Statistical analysis

Continuous variables that follow a normal distribution were presented as mean ± standard deviation; comparisons between two groups were performed using Student’s t-test; and comparisons within multi-groups were performed with one-way ANOVA (Fisher’s LSD test). Continuous variables that follow an abnormal distribution were presented as median and IQR; comparisons between two groups were performed using Wilcoxon’s rank-sum test; and comparisons within multi-groups were performed using Mann–Whitney U-test. Categorical data were expressed in percentage. Comparisons between two groups were performed using Pearson’s chi-square test. Spearman’s rank correlation test was used for correlation analysis. Univariable and multivariable Cox regression analyses were used to evaluate the prognostic factors. Time-dependent receiver operating characteristic (ROC) curves were generated by “survivalROC” in the R package. Nomogram and calibration analyses were carried out by “rms” and “survival” in the R package. The “rmda” in R was used to draw the decision curve analysis (DCA) decision curves. The reported P values were for a two-tailed test. *p* < 0.05 was deemed as statistically significant. Analyses were performed with SPSS (version 23) and R statistical language (version R 4.0.3, https://www.r-project.org/).

## 3 Results

### 3.1 Identification of immune cell infiltration and immune-related genes in MPM

**FIGURE 1:**
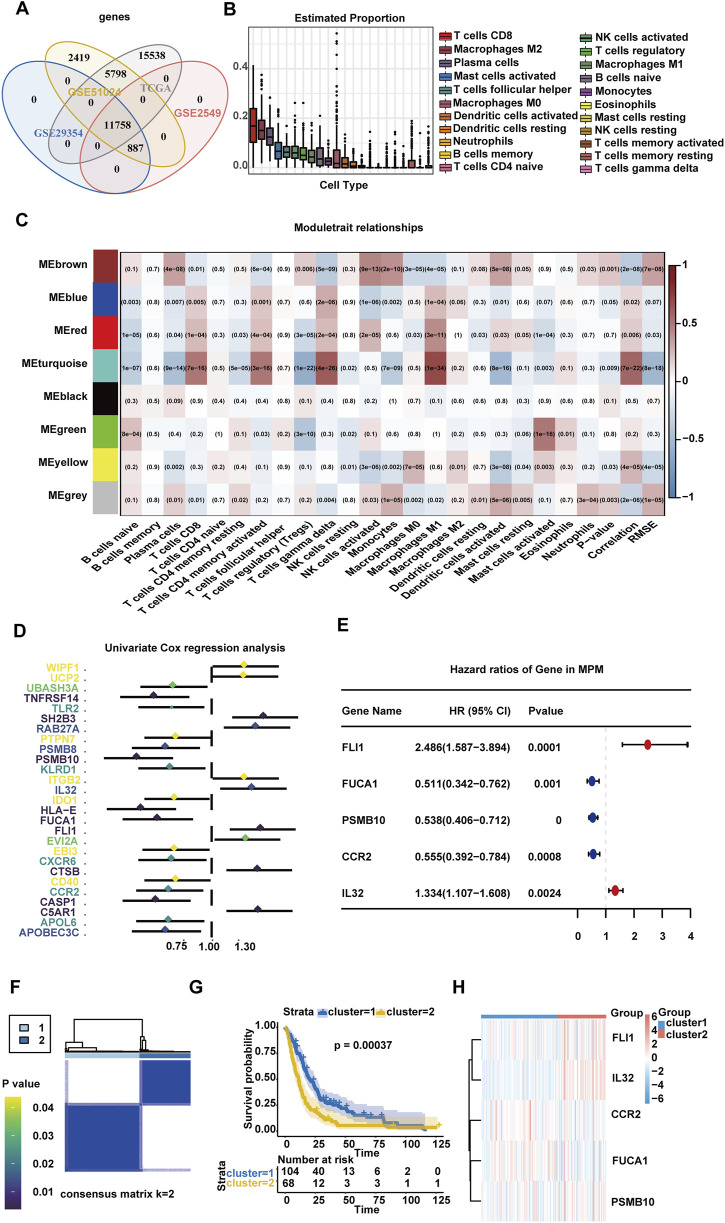
Identification of genes related to the prognosis of MPM in the immune microenvironment. **(A)** Venn diagram of four independent datasets. **(B)** Boxplot of relative infiltration level of immune cells in TCGA and GEO MPM cohorts. **(C)** Gene network visualized via heatmap. **(D)** Univariate Cox regression analysis of immune cell-related genes. **(E)** Hazard ratios and *p* values of multivariate Cox regression for immune-related genes. **(F)** Consistent score matrix for all samples at k = 2 in TCGA and GEO cohorts. **(G)** Overall survival curves for two clusters of 172 patients with MPM from TCGA and GEO cohorts. **(H)** Heat map showing the differential gene expression of five immune-related genes between two MPM subpopulations.

A total of 289 samples of transcriptome data (Supplementary Table S1) from the TCGA and GEO database were analyzed to determine the role of different tumor-infiltrated immune cells in patients with MPM. First, 11,758 co-expressed genes were obtained from gene intersections out of four datasets (Figure 1A). Next, the transcriptome data of four datasets were combined for subsequent analyses after removal of batch effect (Supplementary Figures S1A–C). CIBERSORT was applied to calculate proportions for 22 types of TIICs in each tumor sample. The infiltrated immune cells in the tumor tissues of patients with MPM were mostly CD8^+^ T cells, M2 macrophages, plasma cells, activated mast cells, and T cell follicular helper (Figure 1B; Supplementary Figure S1D). WGCNA revealed eight gene modules of high co-expression with 22 immune cells, among which the MEturquoise module had the strongest correlation with tumor-infiltrated immune cells, and the genes were closely related with macrophages M1 (*p* < 0.001), CD8^+^ T cells (*p* < 0.001), gamma delta T cells (*p* < 0.001), and memory activated CD4^+^ T cells (*p* < 0.001; Figure 1C; Supplementary Figures S1E, F). K–M analysis was conducted for 348 genes in the MEturquoise module to evaluate the correlation between immune cell-related genes and patient prognosis (Supplementary Figure S2). Genes with *p* < 0.05 were further enrolled in the univariate Cox (Figure 1D) and multivariate Cox regression analyses (Figure 1E). Five immune cell-related genes, including FLI1, IL32, FUCA1, CCR2, and PSMB10, which were independently influential on the prognosis of patients with MPM, were screened.

Unsupervised hierarchical clustering analysis was performed on the basis of the above five immune cell-related genes for identification of two different MPM subgroups, cluster1 and cluster2 (Figure 1F). The results of K–M analysis for the two clusters showed that cluster2 was significantly correlated with worsened OS (*p* = 0.00037) (Figure 1G). The expression levels of the five genes in the two clusters are shown in the heatmap (Figure 1H). The immune microenvironment characteristics of the two clusters were evaluated. The results showed that higher counts of immunosuppressive factors were seen in cluster2 than in cluster1 (Supplementary Figure S3A). The ESTIMATE algorithm revealed that cluster2 scored higher in stroma and immunity (Supplementary Figure S3B). The MSI states of two MPM clusters were not significantly different (*p* = 0.240, Supplementary Figure S3C). The TMB stage of cluster2 was remarkably higher than that of cluster1 (Supplementary Figure S3D). The waterfall plot displayed mutations of somatic cells in cluster1 and cluster2 patients. The enriched gene mutations of the two clusters were not relatively identical (Supplementary Figure S3E). The mutation rates of the five genes were not high, suggesting that mutations were not a primary cause for change in gene expression. At this point, five immune cell-related genes, which can classify patients with MPM into two subgroups, were initially screened. The cluster2 patients demonstrated worse prognosis and more expression of immunosuppressive factors in the TME.

### 3.2 Construction of a prognostic model of MPM based on immune cell-related genes

**FIGURE 2:**
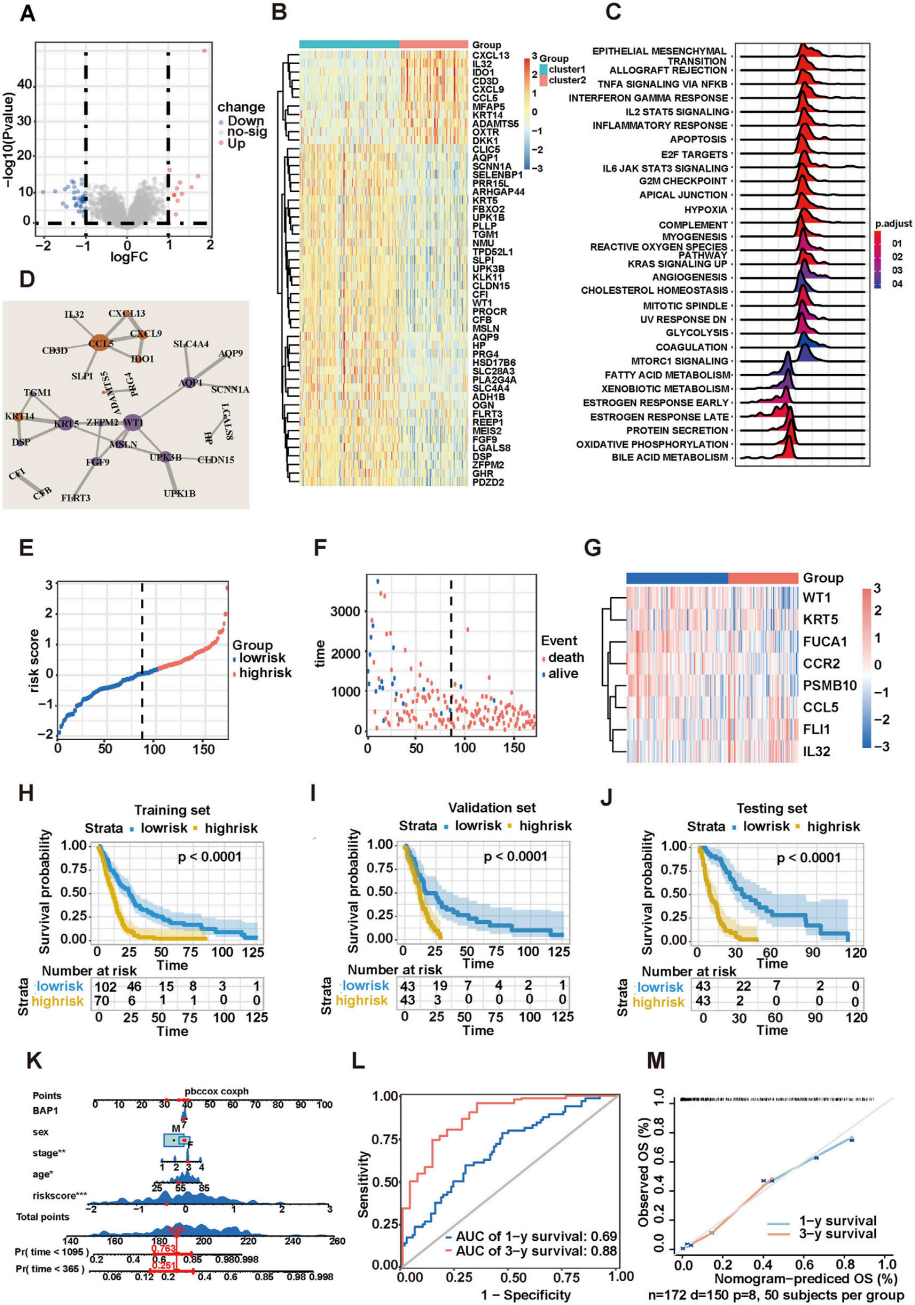
Construction of a risk score model for MPM. **(A)** Volcano map showing 51 differential genes. **(B)** Heatmap showing 51 genes significantly positively and negatively correlated with two MPM subpopulations. **(C)** GSEA enrichment plot of two MPM subpopulations. **(D)** Top three hub genes identified from PPI networks. **(E)** Gene signature risk score distribution. **(F)** Vital status of high- and low-risk groups. **(G)** Heatmap of the expression profiles of members in the risk score model. **(H–J)** Kaplan–Meier survival curves plotted to estimate the overall survival probabilities for the low-risk versus high-risk groups in training, validation, and test sets. **(K)** Nomogram predicting the 1-year and 3-year overall survival of patients with MPM. **(L)** Time-dependent ROC curves plotted for 1-year and 3-year overall survival. **(M)** Calibration curve for the overall survival nomogram model in TCGA and GEO MPM cohort. The dashed diagonal line represents the ideal nomogram, and the blue line and red line represent the 1-year and 3-year observed nomograms, respectively.

Differential gene enrichment analysis was performed to identify differences in patients with two different immune microenvironment statuses. A total of 51 significant DEGs were obtained after differential gene analysis of cluster1 and cluster2, among which 11 genes were upregulated and 40 genes were downregulated (Figure 2A). Figure 2B displays the expression levels of 51 DEGs in cluster1 and cluster2. GO analysis and GSEA were performed to determine the DEG function. The five highest ranking GO terms of BP, MF, and CC are shown in Supplementary Figure S3F. The receptor–ligand activities, signaling receptor activator, and chemokine pathways were significantly activated. BP ontology showed that DEGs were concentrated in the cellular response pathway to fibroblast growth factor. The GSEA analysis revealed a significant increase of signaling pathways in cluster2 related to cell metastasis and inflammatory responses, such as EMT, G2M checkpoint, interferon gamma response, and apoptosis (Figure 2C; Supplementary Figure S3G). A PPI network was constructed to identify key genes associated with cluster1 and cluster2 phenotypes. The former three hub genes in the critical node, which were extensively connected with many DEGs, were CCL5, WT1, and KRT5 (Figure 2D).

Five immune cell-related genes and three hub genes were combined to construct an eight-gene risk score model. The threshold value between dead patients and live patients (cutoff = 0.204,853) was obtained by drawing the ROC curve and determining the risk scores with the maximum Youden index, based on which patients were divided into low-risk group (n = 102) and high-risk group (n = 70, Figure 2E). As the risk scores increased, the death ratio of patients with MPM increased with a shortened OS (Figure 2F). Among the eight genes used to construct the risk score model, IL-32, CCL5, and FLI1 were significantly upregulated in high-risk patients, whereas CCR2, PSMB10, KRT5, WT1, and FUCA1 demonstrated low expression levels (Figure 2G). The survival analysis showed that the survival probability of the high-risk group was significantly lower than that of the low-risk group (*p* < 0.0001, Figure 2H). The entire dataset was randomly divided at a 1:1 ratio into validation set (n = 86) and test set (n = 86) for K–M survival analysis. As expected, the prognosis of patients with high-risk scores was significantly worse (Figures 2I,J). These data indicated that the features of eight genes in the model contribute to MPM prognostic prediction.

### 3.3 Eight-gene risk score model being an independent predictor of MPM

Risk scoring, TNM stage, BAP1 gene status, age, and gender were included in the multivariate Cox regression analysis to confirm whether risk scoring could be used as an independent predictor for OS among patients with MPM. Age, TNM stage, and risk scoring were found to have independent prognostic meaning (Supplementary Figure S3H). On the basis of these findings, a nomogram available in clinical practice was established for clinicians to predict the prognosis of patients with MPM with a better quantitative approach. The survival probabilities of patients at first and third years were evaluated (Figure 2K). The predictive ability of the nomogram was determined using time-dependent ROC curves (Figure 2L). The results showed that the predictive accuracy of the nomogram gradually increased with the extension of OS. Meanwhile, a calibration curve was constructed, and the results indicated that the predictive and actual rates of survival were consistent at first and third year (Figure 2M). Finally, DCA demonstrated that the nomogram risk scoring was capable of evaluating the OS of patients with MPM accurately (Supplementary Figure S3I). Patients were stratified in accordance with TNM stages to further verify the universality of risk scoring in MPM, and the OS of patients with high- and low-risk scores were compared. The results showed that regardless of the stage, patients with MPM with higher risk scores were associated with worse prognosis (Supplementary Figures S3J,K).

A survival analysis of patients in the high- and low-risk groups involving 32 types of tumors in TCGA other than MPM was performed to further validate the performance of risk score in predicting prognosis of other tumors (Supplementary Figure S4A). Patients in the low-risk group had a significant survival advantage in 24 tumors, including adrenocortical carcinoma (ACC, *p* = 0.00012), bladder urothelial carcinoma (BCLA, *p* = 0.0013), breast invasive carcinoma (BRCA, *p* < 0.0001), cervical squamous cell carcinoma and endocervical adenocarcinoma (CESC, *p* = 0.021), glioblastoma multiforme (GBM, *p* = 0.0014), esophageal carcinoma (ESCA, *p* = 0.0046), lymphoid neoplasm diffuse large B-cell lymphoma (DLBC, *p* = 0.012), colon adenocarcinoma (COAD, *p* = 0.04), kidney renal clear cell carcinoma (KIRC, *p* < 0.0001), kidney renal papillary cell carcinoma (KIRP, *p* = 0.00012), acute myeloid leukemia (LAML, *p* = 0.0007), brain lower grade glioma (LGG, *p* < 0.0001), ovarian serous cystadenocarcinoma (OV, *p* = 0.0016), lung squamous cell carcinoma (LUSC, *p* = 0.0007), lung adenocarcinoma (LUAD, *p* < 0.0001), pheochromocytoma (PCPG, *p* = 0.028), sarcoma (SARC, *p* = 0.00017), skin cutaneous melanoma (SKCM, *p* < 0.0001), uterine corpus endometrial carcinoma (UCEC, *p* = 0.00049), thymoma (THYM, *p* = 0.0016), thyroid carcinoma (THCA, *p* = 0.014), stomach adenocarcinoma (STAD, *p* = 0.0016), uterine carcinosarcoma (UCS, *p* = 0.049), and uveal melanoma (UVM, *p* < 0.0001). The predictive performance of the risk score in pan-cancer level was displayed by ROC curve, and the AUC values are presented in Supplementary Figure S4B. In addition to MPM, the risk score model demonstrated good prognostic predictive power for pan-cancer species.

### 3.4 Low-risk group being more likely to benefit from immunotherapy and chemotherapy

**FIGURE 3:**
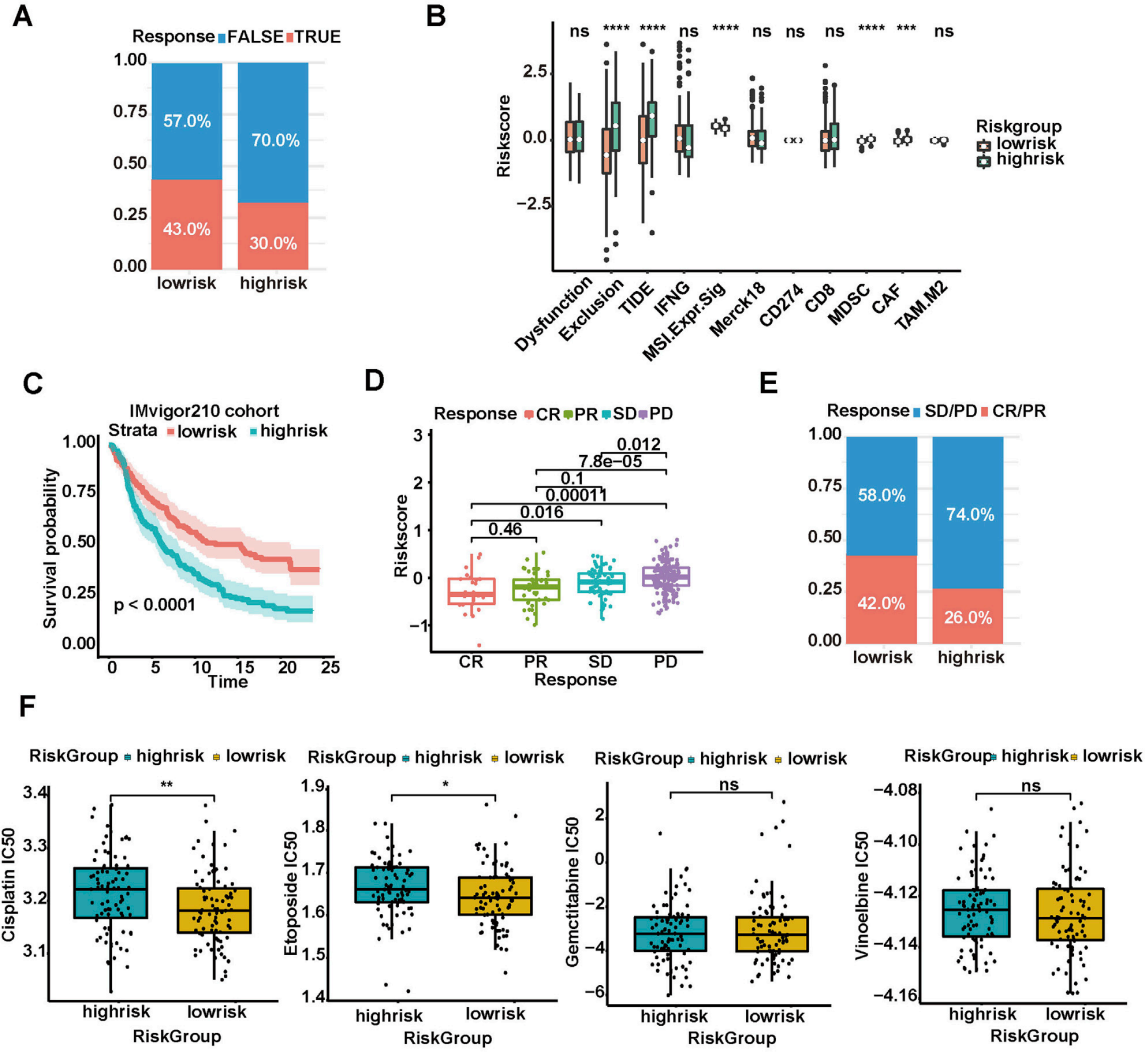
Prediction of tumor response to immunotherapy and chemotherapy based on risk score model. **(A)** Rate of clinical response (TRUE and FALSE) to immunotherapy in high or low-risk groups of 289 MPM in TIDE database. **(B)** TIDE database-evaluated scores of immunosuppressive cells and scores of T cell dysfunction and exclusion in diverse risk groups. **(C)** Kaplan–Meier survival curves plotted to estimate the overall survival probabilities for the low-risk versus high-risk group in IMvigor210 cohort. **(D)** Different anti-PD-L1 clinical response status groups (CR, complete response; PR, partial response; PD, progressive disease; SD, stable disease). **(E)** Rate of clinical response (CR/PR and SD/PD) to immunotherapy in high- or low-risk groups. **(F)** Boxplots showing the IC50s of four common chemotherapeutic agents (cisplatin, etoposide, gemcitabine, and vinorelbine) in high- and low-risk patients with MPM (*p < 0.05, **p < 0.01, ***p < 0.001, ****p < 0.0001).

The immunotherapeutic response and T cell functional status for patients with MPM were indirectly predicted by adopting the NSCLC immunotherapy cohort in TIDE database. The low-risk group had a higher ratio of response to immunotherapy (43% vs 30%, Figure 3A). The low-risk group also scored lower in TIDE than the high-risk group, suggesting that the low-risk group was more likely to benefit from immunotherapy (Figure 3B). Thus, the patients had a significant rejection feature of enriched T-cells due to higher infiltration of myeloid-derived suppressor cells (MDSCs) and tumor-associated fibroblasts (CAFs) in the high-risk group. The validity of risk score in the immunotherapy cohort for treating advanced urothelial carcinoma (IMvigor210 cohort) with atezolizumab (anti-PD-L1 antibodies) was indirectly verified. The results showed the worse prognosis of patients with high-risk scores than those with low-risk scores, of which both received immunotherapy (Figure 3C). A correlation was found between the risk score and response to immunotherapy. The risk scores of patients with response to immunotherapy [complete response (CR) + partial response (PR)] were significantly lower than those of patients with poor response [stable disease (SD) + progressive disease (PD)], Figure 3D). The low-risk group had a higher proportion of objective response (CR + PR), which was consistent with the results in TIDE database (42% vs 26%, Figure 3E).

By using the “pRRophetic” algorithm, the IC50s of four conventional chemotherapeutic agents (cisplatin, etoposide, gemcitabine, and vinorelbine) in high-risk and low-risk patients with MPM were predicted. The results showed that the IC50s of all four chemotherapeutic agents in the high-risk group increased, and the differences were statistically significant in cisplatin and etoposide, indicating that patients in the high-risk group were less sensitive to these two agents (Wilcoxon test, *p* < 0.01; Figure 3F). Thus, the risk score models can be used to predict the efficacy of immunotherapy and chemotherapy.

### 3.5 FUCA1 expression pattern and its correlation with prognosis in MPM

**FIGURE 4:**
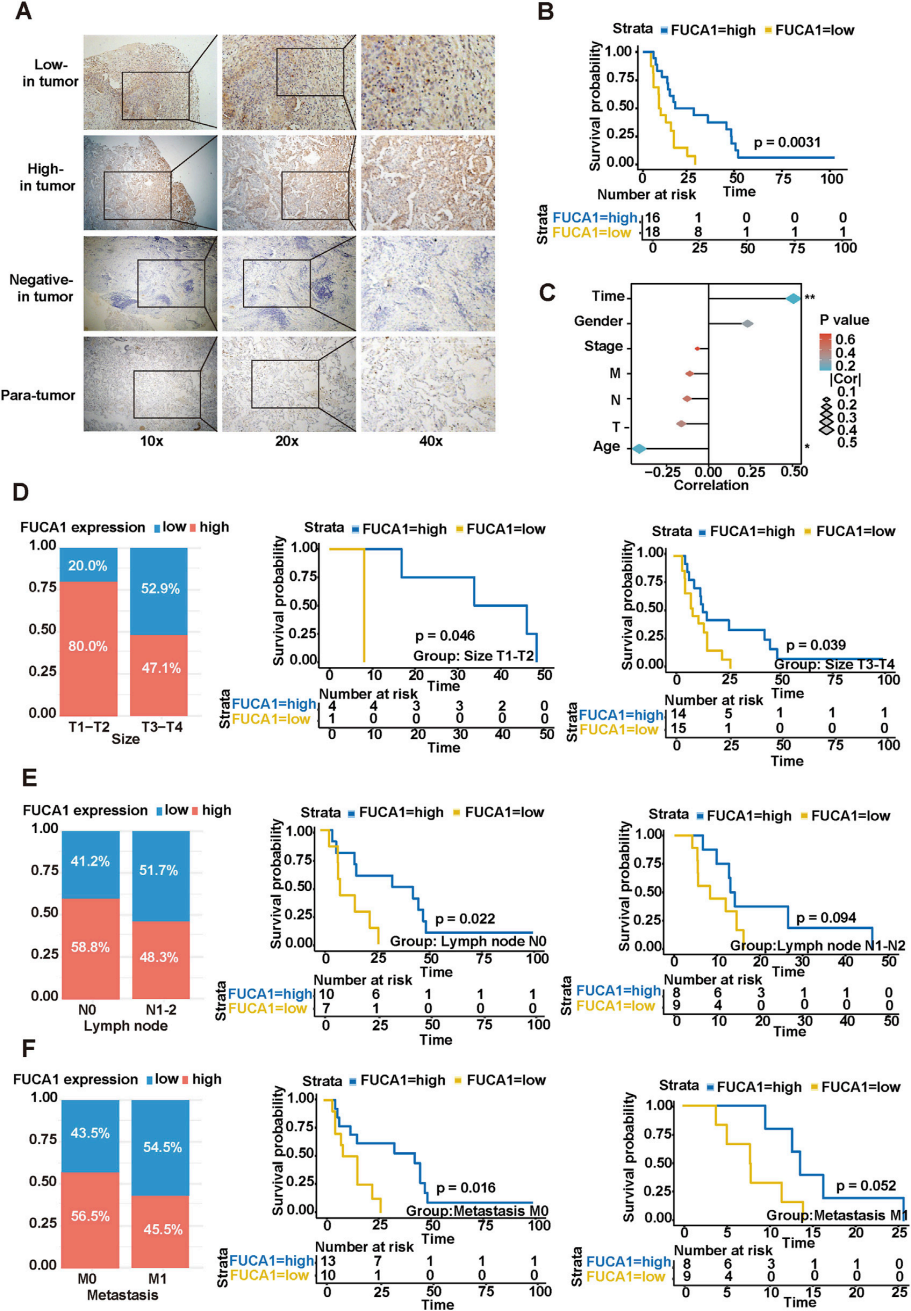
Correlation of FUCA1 with clinicopathological features of patients with MPM. **(A)** Different expression levels of FUCA1 in malignant pleural mesothelioma cancer tissue and paracancerous tissue samples. **(B)** Correlation between FUCA1 expression level and prognosis of patients with pleural mesothelioma. **(C)** Correlation analysis between FUCA1 expression and different risk factors. **(D)** Relationship between FUCA1 expression and OS in tumor size subgroups. **(E)** Relationship between FUCA1 expression and OS in a subgroup of lymph node metastases. **(F)** Relationship between FUCA1 expression and OS in a subgroup of distant metastases. (*p < 0.05, **p < 0.01).

FUCA1 was chosen for further study because it had the greatest correlation with the risk score and the lowest HR in the multivariate analysis, leading to the highest contribution to the predictive model (Figure 1E; Supplementary Table S3). In addition, the functions of FUCA1 in MPM remain unclear. The K–M survival analysis and violin diagram showed that patients with low FUCA1 expression had a worse prognosis (Supplementary Figures S5A, B). This finding was validated from this study’s MPM cohort to further probe the expression pattern of FUCA1 in MPM. Formalin-fixed paraffin-embedded tumor samples and complete medical records of 34 patients with MPM from Tianjin Medical University Cancer Institute and Hospital between January 2013 and July 2021 were collected. Figure 4A demonstrates the different expression levels of FUCA1 in cancer and paracancerous tissues of patients with MPM. The K–M survival analysis result was consistent with the previous prediction (Figure 4B). Distant metastasis, TNM stage, and FUCA1 expression were independent risk factors for prognosis in patients with MPM (Supplementary Table S4). This series of results indicated that FUCA1 could be used as a specific biomarker in patients with MPM. Next, the associations of FUCA1 expression with various clinicopathological factors were assessed. The expression of FUCA1 was closely correlated with patient survival time and age (Figure 4C). Four out of five (80.0%) cases with a tumor size T1-T2 showed high FUCA1 expression. By contrast, 14 out of 29 (48.3%) cases with a tumor size T3-T4 exhibited high FUCA1 expression (Figure 4D). Furthermore, 10 out of 17 (58.8%) cases with no lymphatic metastasis N0 showed high FUCA1 expression. Eight out of 17 (47.1%) cases with lymphatic metastasis N1-N2 exhibited high FUCA1 expression (Figure 4E). Thirteen out of 23 (56.8%) cases with no distant metastasis M0 showed high FUCA1 expression. Five out of 11 (45.5%) cases with distant metastasis M1 exhibited high FUCA1 expression (Figure 4F). Similarly, in different TNM stage subgroups, the patients with high FUCA1 expression could still achieve longer OS than those in the low expression cohort (*p* = 0.0042, *p* = 0.034, Supplementary Figure S5C). Thus, the results clearly indicated that FUCA1 is expressed at low levels in MPM tissues, and low FUCA1 expression correlates with poor prognosis. Decreased FUCA1 expression may play an important role in MPM progression and recurrence.

### 3.6 Promotion of MPM cell proliferation, migration, and invasion by knockdown of FUCA1

**FIGURE 5:**
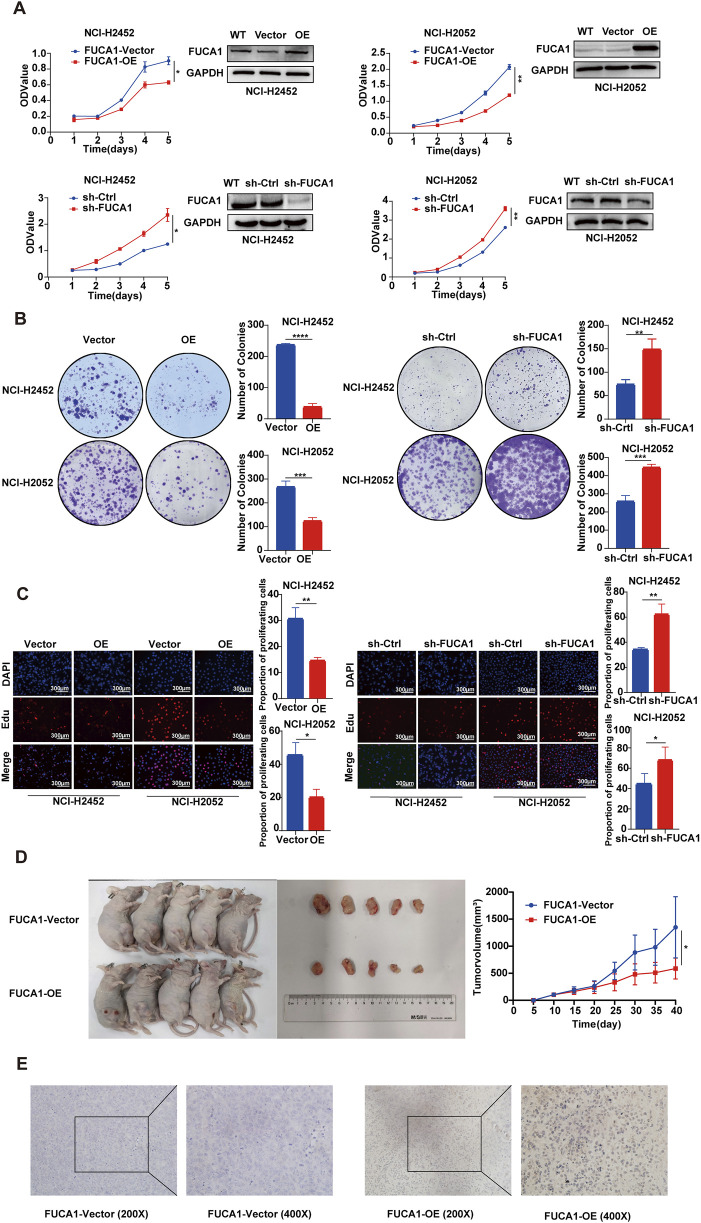
FUCA1 inhibition of MPM cell proliferation. **(A–C)** Verification of FUCA1 expression by using WB after transfection. In NCI-H2452 cells and NCI-H2052 cells, CCK8 assay, cloning formation assay, and EdU proliferation assay indicated that FUCA1 expression was inversely related to cell proliferation ability. **(D)** In BALB/c nude mice, the tumor size was smaller in the FUCA1-OE group than in the FUCA1-vector group. **(E)** Representative images of the expression level of FUCA1 in mouse tumor tissues. (*p < 0.05, **p < 0.01, ***p < 0.001, ****p < 0.0001)

**FIGURE 6:**
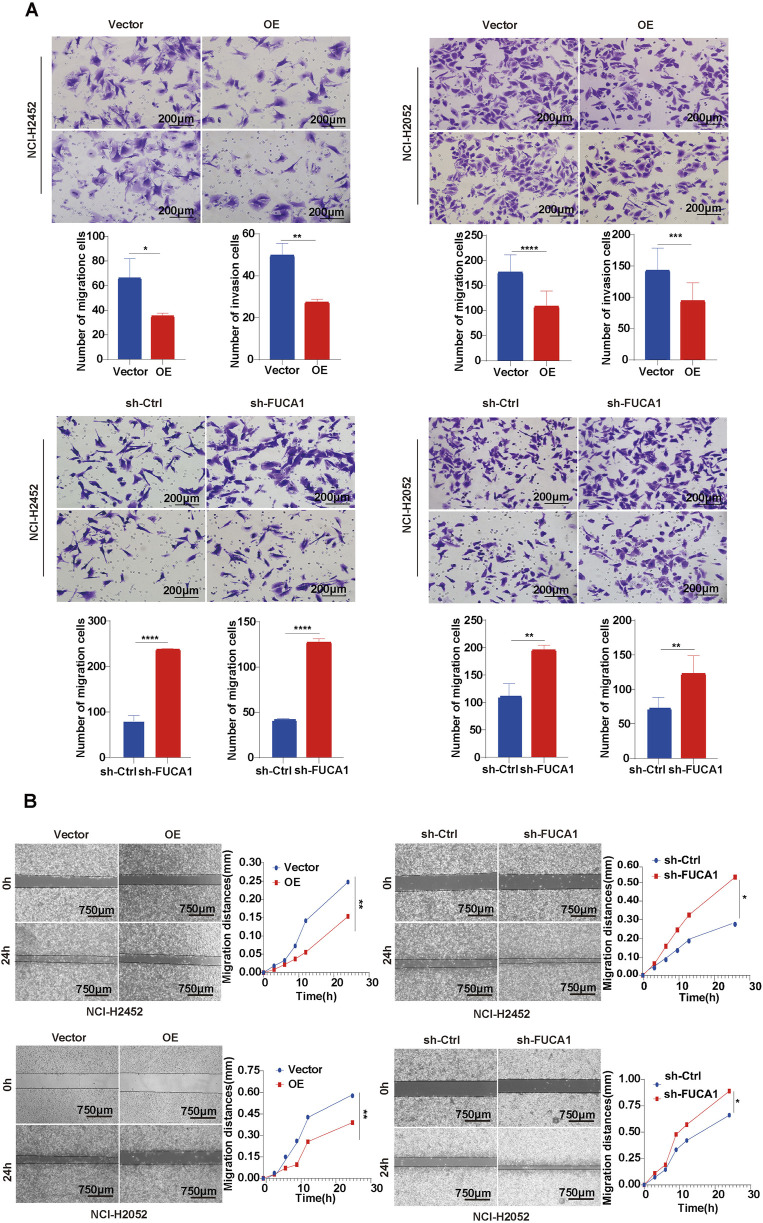
FUCA1 inhibition of MPM cell invasion and migration. **(A)** Transwell assay for evaluation of cell invasive ability. **(B)** Wound healing assay for analysis of cell migration ability. (*p < 0.05, **p < 0.01, ***p < 0.001, ****p < 0.0001).

Next, we further explored the function of FUCA1 in MPM through a series of *in vitro* and *in vivo* experiments. Firstly, the FUCA1 expression in BEAS-2B, NCI-H2452, and NCI-H2052 cells was examined (Supplementary Figure S5D). Next, NCI-H2452 and NCI-H2052 cells were used to establish stable overexpression and knockdown cell lines. The control cells Vector and sh-Ctrl were constructed. The efficiency of FUCA1 deletion and overexpression was confirmed by WB and real-time PCR (Figure 5A; Supplementary Figure S5E). Then, the proliferation of MPM cells with different expression levels of FUCA1 was evaluated using CCK-8 assay (Figure 5A), colony formation assay (Figure 5B), EdU proliferation assay (Figure 5C), and vivo mouse experiments (Figure 5D). The results showed that the FUCA1-knockdown group had a stronger proliferative ability, and the proliferation ability was decreased after the overexpression of FUCA1. We verified the expression level of FUCA1 in mouse tumour tissues using immunohistochemistry (Figure 5E). The results of Transwell assay (Figure 6A) and wound-healing assay (Figure 6B) in NCI-H2452 and NCI-H2052 cells showed that FUCA1 knockdown promoted cell invasion and migration when the upregulation of FUCA1 suppressed the migration and invasion abilities *in vitro*. Therefore, the decrease in FUCA1 expression induced the proliferation, invasion, and metastasis of MPM cells, consistent with the previous analysis of clinical data.

### 3.7 FUCA1 regulation of EMT through PI3K-AKT signaling pathway

**FIGURE 7:**
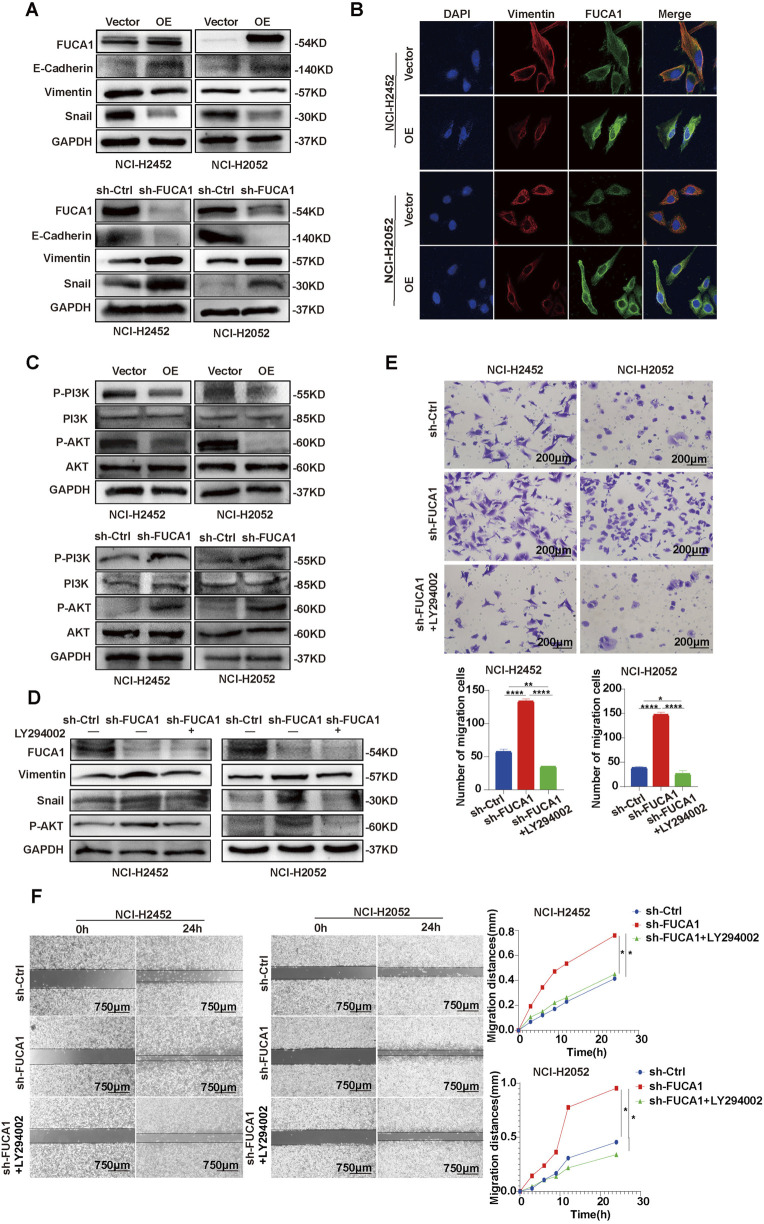
FUCA1 regulation of EMT in MPM cells through PI3K-AKT signaling pathway. **(A)** WB demonstrating that FUCA1 expression was negatively correlated with EMT. **(B)** Immunofluorescence confirmation that overexpression of FUCA1 suppressed the expression levels of mesenchymal phenotype genes. **(C)** WB showing that FUCA1 inhibited PI3K-AKT signaling pathway. **(D)** WB showing that LY294002 inhibited EMT in FUCA1 knockdown cells. **(E)** LY294002 inhibition of the invasive capacity of FUCA1 knockdown cells. **(F)** LY294002 inhibition of the migration capacity of FUCA1 knockdown cells. (*p < 0.05, **p < 0.01, ***p < 0.001, ****p < 0.0001).

In the preliminary analysis, GSEA showed that FUCA1 expression was negatively correlated with EMT in MPM (Figure 2C; Supplementary Figure S3G). Next, the mRNA expression of FUCA1 in EMT-associated genes was analyzed using the ENCORI database. FUCA1 was positively correlated with the epithelial phenotypes E-Cadherin, OCL, and TJP2 and negatively correlated with the mesenchymal phenotypes vimentin, CDH2, ZEB1, SNAL1, SNAL2, TWIST1, and FN1 (Supplementary Figure S6A). WB was used to verify that the knockdown of FUCA1 decreased E-Cadherin and increased vimentin and snail in NCI-H2452 and NCI-H2052 cells. FUCA1 overexpression showed the opposite effects on these proteins in NCI-H2452 and NCI-H2052 cells (Figure 7A; Supplementary Figure S7A). Immunofluorescence was used to verify that the knockdown of FUCA1 increased vimentin (Figure 7B). All these results suggested that the knockdown of FUCA1 promoted EMT, and that FUCA1 may regulate the malignant phenotypic transformation of MPM cells. KEGG software was used to explore some related signaling pathways to demonstrate further the downstream molecular mechanism of FUCA1. Pathway enrichment was performed on the basis of DEGs. Genes in the PI3K-AKT signaling pathway were significantly enriched (Supplementary Figure S6B). Next, WB revealed that downregulated FUCA1 expression strengthened the protein levels of p-PI3K and p-AKT in NCI-H2452 and NCI-H2052 cells. The p-PI3K and p-AKT protein expression levels decreased when FUCA1 was overexpressed. However, the protein levels of PI3K and AKT did not change significantly (Figure 7C; Supplementary Figure S7B). NCI-H2452 and NCI-H2052 FUCA1 knockdown cells were treated with LY294002, an PI3K-AKT inhibitor, to investigate the relationship between the PI3K-AKT pathway and FUCA1. p-AKT expression was significantly increased in sh-FUCA1 cells and effectively suppressed upon treatment with LY294002. WB further showed that the LY294002-mediated inhibition of p-AKT significantly attenuated the levels of vimentin and snail in sh-FUCA1 cells (Figure 7D; Supplementary Figure S7C). Besides, the Transwell assay (Figure 7E) and wound-healing assay (Figure 7F) showed that the LY294002-mediated inhibition of p-AKT significantly suppressed the invasion and migration in sh-FUCA1 cells. All these results indicated that FUCA1 regulated EMT in MPM cells through the PI3K-AKT signaling pathway.

## 4 Discussion

MPM is an uncommon but aggressive tumor disease that has been unequivocally linked to asbestos exposure. This association underscores the critical need for effective preventive measures and early detection strategies to mitigate the risk of developing this disease. The TIME of MPM is highly heterogeneous, which is generally considered as immune tolerance or immunosuppression. This is one of the reasons why most of patients with MPM cannot benefit from immunotherapy. An enhanced understanding of this TIME may help screen out sensitive population to the treatment and develop new treatment strategies. However, in the highly complex microenvironment of solid tumors, single biomarkers fail to satisfy clinical requirements. Some gene panels and algorithms based on immune cells and immune functions has certain reference value in predicting the efficacy of immunotherapy (Ayers et al., 2017; Damotte et al., 2019).

By integrating expression data from multiple datasets, an eight-gene risk score model that successfully divided patients with MPM into low- and high-risk groups was constructed. Notably, the high-risk group exhibited a survival disadvantage, and they were less sensitive to immunotherapy, indicating that the model could be a valuable tool for predicting immunotherapy outcomes. Among the eight genes included in the model, FUCA1 emerged as a key player. The clinical data indicated that FUCA1 is an independent risk factor for predicting the prognosis of patients with MPM, suggesting its potential as a prognostic biomarker. This finding is particularly remarkable given the lack of good biomarkers for MPM.

At present, immunotherapy has become a new strategy for cancer treatment, and in fact, only about 20% of patients with solid tumors could benefit from such therapies (Braun et al., 2016). Especially for patients with MPM with highly heterogeneous TIME, identifying and validating the indicators that can accurately predict efficacy of immunotherapy are particularly important. The focus of previous tumor studies had always been on deciphering the intrinsic characteristics of tumor cells. In recent years, spotlight has gradually been shifted to the concept of interactions among tumor cells, immune cells, and other cell types. In TME, the interactions between tumor cells and immune cells have changed in the between balance, which tends to activate tumor survival and promote tumor immune evasion (Kersten et al., 2015), which, in turn, promotes tumor progression and metastasis. A tumor growth-promoting microenvironment was formed by producing various cytokines and enzymes that promote tumor proliferation and metastasis, angiogenesis, and immunosuppression (Yang et al., 2020). In the enrichment analysis, the gene-related pathways of high-risk group were mostly associated with pro-tumor metastasis, proliferation, and pro-inflammatory reactions. Meanwhile, the level of immunosuppression factors in tumor tissues, such as LAG-3, CTLA-4, and IDO-1, increased significantly.

FUCA1 encodes the tissue α-L-fucosidase (Intra et al., 2007), a lysosomal enzyme involved in the degradation of L-focusing residues at the end of oligosaccharide chains in glycoconjugates. In tumor biology, the aberrant glycosylation of tumor cells reflects specific modifications of their glucose metabolic pathways. α-L- fucosidase is involved not only in the development of various malignant tumors but also in the regulation of immune escape, invasion, and metastasis of tumors. Previous studies have shown that low FUCA1 expression is associated with worsened prognosis in thyroid (Tsuchida et al., 2017), colon (Otero-Estévez et al., 2013), and breast cancers (Cheng et al., 2015). The tumor suppressor p53 is known to be a key regulator of programmed cell death (Vogelstein et al., 2000), and p53 can activate FUCA1 transcriptionally (Tsuchida et al., 2017). Expression of FUCA1 induced apoptosis in renal tumor cells, and knockdown of FUCA1 enhanced the proliferation of lung cancer cells (Ezawa et al., 2016). In lung cancer, FUCA1 can inhibit EGFR signaling and its downstream signaling by inhibiting AKT phosphorylation (Ezawa et al., 2016). However, downregulation of FUCA1 expression inhibited glioma progression by enhancing autophagy and suppressing macrophage infiltration (Xu et al., 2020). A series of experiments was conducted to further investigate the role of FUCA1 in MPM. The results demonstrated that FUCA1 expression was negatively correlated with the proliferation, invasion, and migration abilities of MPM cells, suggesting that FUCA1 may have a tumor-suppressive role in MPM.

Furthermore, the expression of FUCA1 was negatively correlated with EMT in MPM cells by regulating the PI3K-AKT signaling pathway. EMT is a process of epithelial-to-mesenchymal cell transformation in which epithelial cells lose acinar cell polarity, lose adhesion and acquire a mesenchymal cell phenotype, and gain mesenchymal cell migration capacity to facilitate metastasis and drug resistance (Thiery, 2002). Tumor cell invasion and migration are important biological processes in tumor development, and tumor metastasis is an important cause of tumor progression. An increasing number of experiments have confirmed that the EMT process plays an important role in the invasion and metastasis of solid tumor cells (Thiery et al., 2009). The occurrence of EMT in cancer cells can cause immune escape, so patients become insensitive to anti-CTLA-4 therapy (Dongre et al., 2021). Decreased expression of FUCA1 in bladder epithelial cells contributed to increased expression of fucosylated N-glycans in TGF-β-induced EMT (Guo et al., 2014). Previous studies reported that FUCA1 may inhibit the PI3K-AKT pathway activation through glycosylation modification (Hu et al., 2024; Zhuang et al., 2024). The present study confirmed that FUCA1 may contribute to the suppression of MPM cell proliferation, invasion, and migration by inhibiting EMT.

The risk score model provides a new perspective for screening potential populations that may benefit from immunotherapy and predicting their efficacy. Clinicians can tailor treatment strategies to improve outcomes by identifying high-risk patients. The findings suggest that FUCA1 may be a promising therapeutic target for patients with MPM. Targeting FUCA1 could potentially inhibit cancer progression and improve patient prognosis.

However, a notable detail that this study has limitations. First, this study assessed the predictive power of risk score models for immunotherapy outcomes by using an immunotherapy cohort of patients with NSCLC and patients with uroepithelial carcinoma due to the lack of data on immunotherapy in patients with MPM. Second, the sample size was relatively small, and the findings may not be generalizable to all patients with MPM. Further validation in larger and more diverse patient cohorts is needed to confirm the robustness of the risk score model and the prognostic value of FUCA1.

## 5 Conclusion

This study, conducted from a multi-omics perspective, found that immune cell-related genes are informative in predicting the survival prognosis and efficacy of immunotherapy in patients with MPM. The risk score model constructed in this study provides a new perspective for screening potential populations to benefit from immunotherapy and predicting their survival. FUCA1 may be a potential prognostic biomarker and promising therapeutic target for patients with MPM.

## Data Availability

The original contributions presented in the study are included in the article/Supplementary Material, further inquiries can be directed to the corresponding authors.
