# Supplementary material for: Immune-related gene risk model establishment and role of key gene FUCA1 in malignant pleural mesothelioma
Source: Front Pharmacol. 2025 May 23;16:1577232. doi: 10.3389/fphar.2025.1577232 (PMC12141270; doi:10.3389/fphar.2025.1577232)
Supplement: Supplementary file 1 [file DataSheet1.docx]

Supplementary Material

# Supplementary Tables


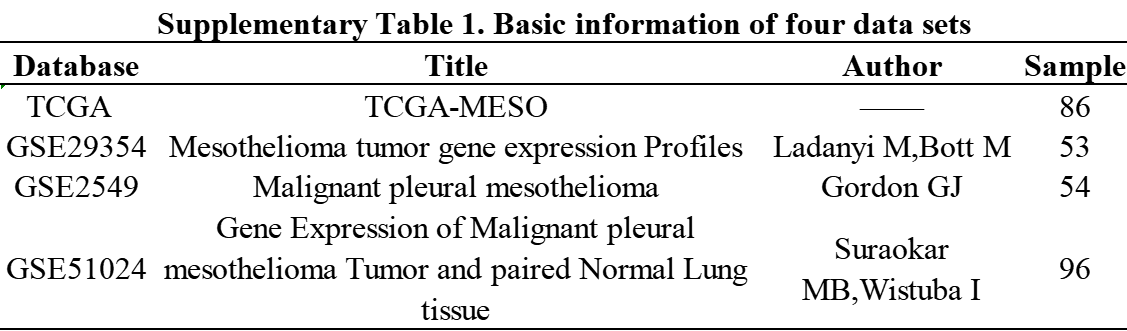


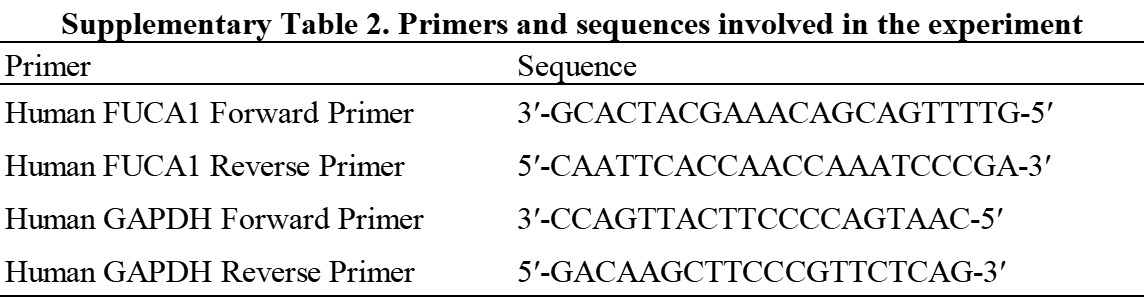


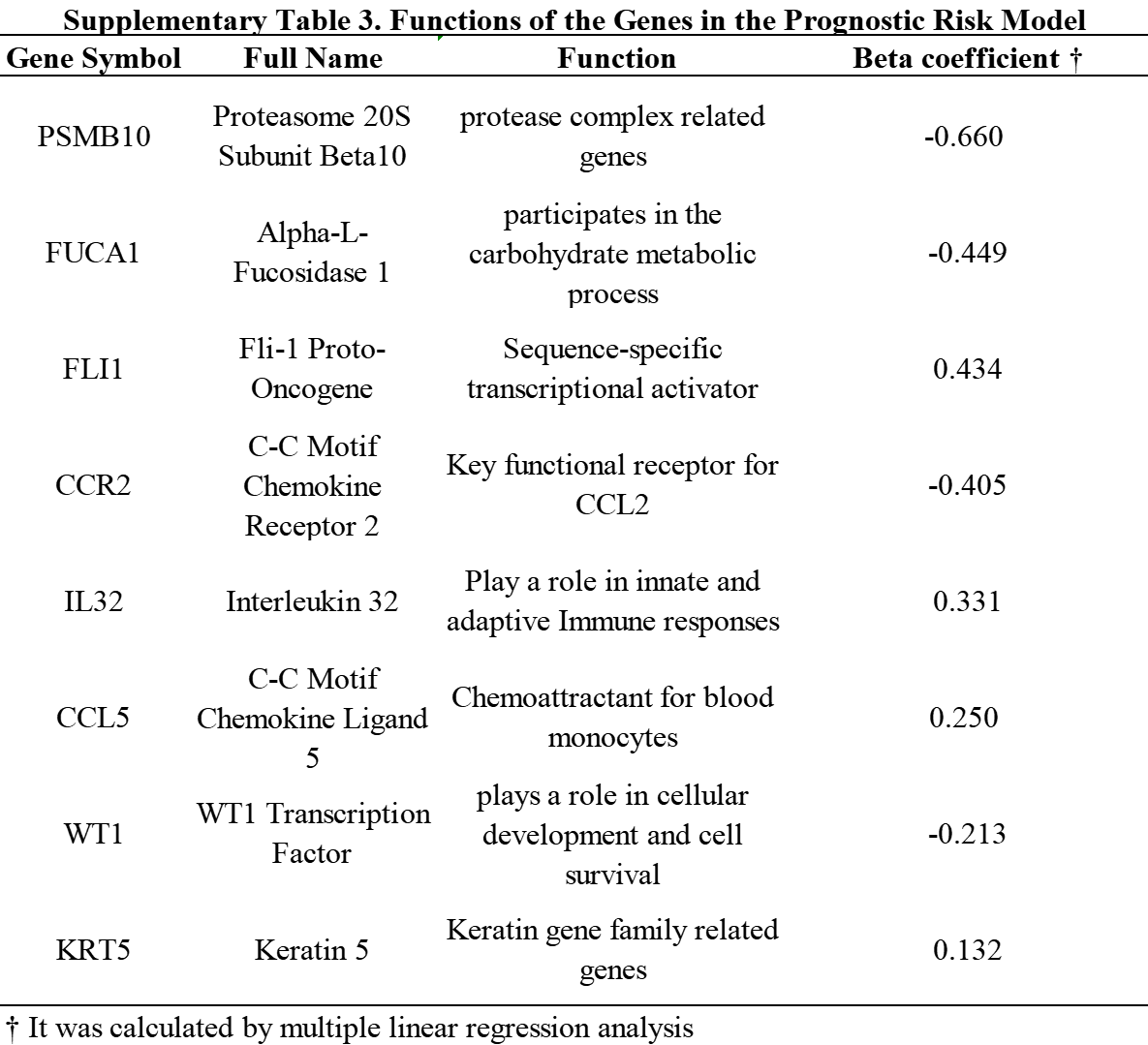


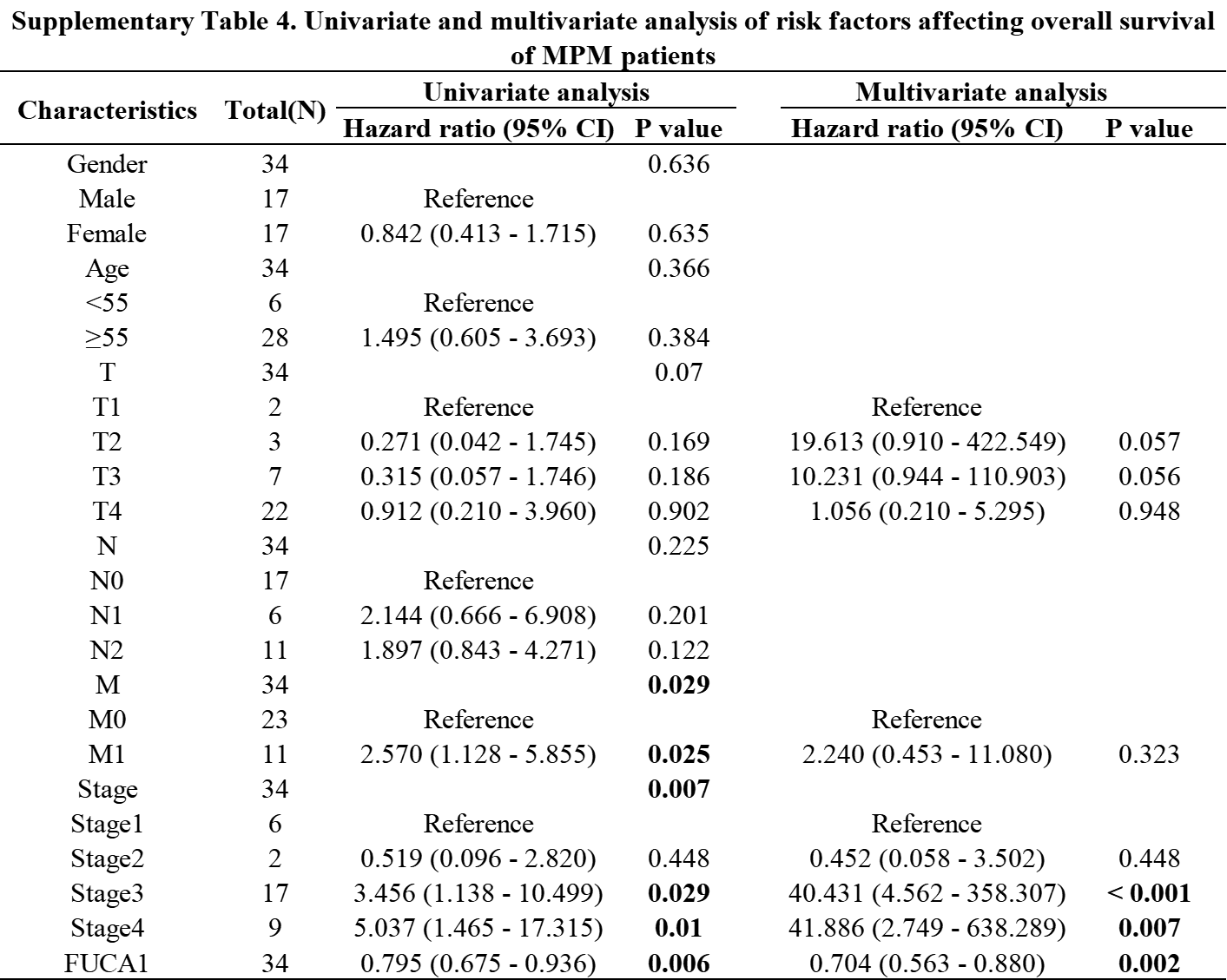


# Supplementary Figures
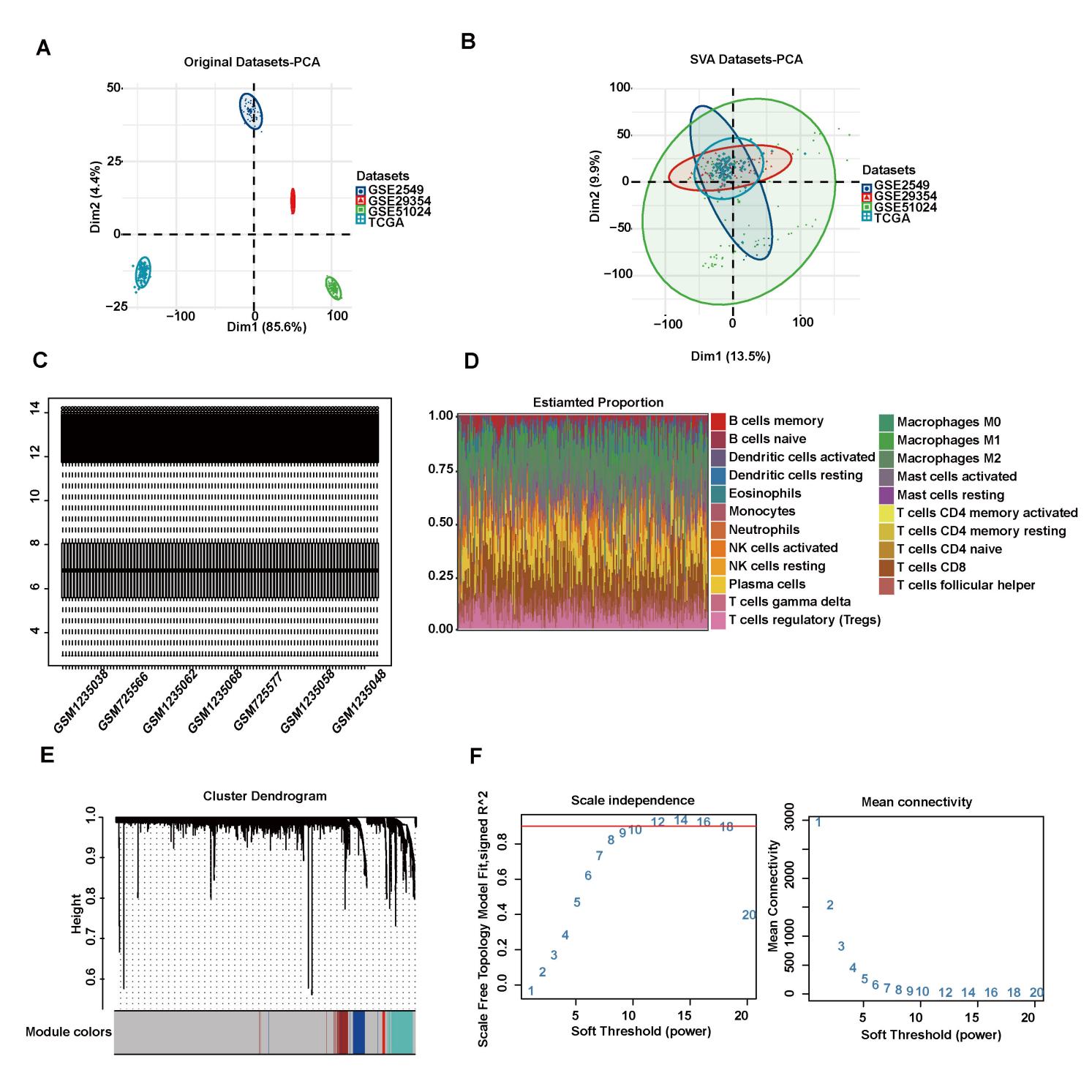


**Supplementary Figure 1: Identification of immune-related genes in MPM.** **(A, B)** Four independent datasets before and after correction for de-batch effects. **(C)** Boxplot showing the MPM sample after correcting for batch effects. **(D)** Distribution of 22 immune cells in malignant pleural mesothelioma samples from TCGA and GEO datasets. **(E)** Dissimilarity gene clustering dendrogram based on topological overlap with specified module colors. **(F)** Moules defined by adopting the layered clustering method with weighted coefficient matrices. Soft threshold power value b = 9.


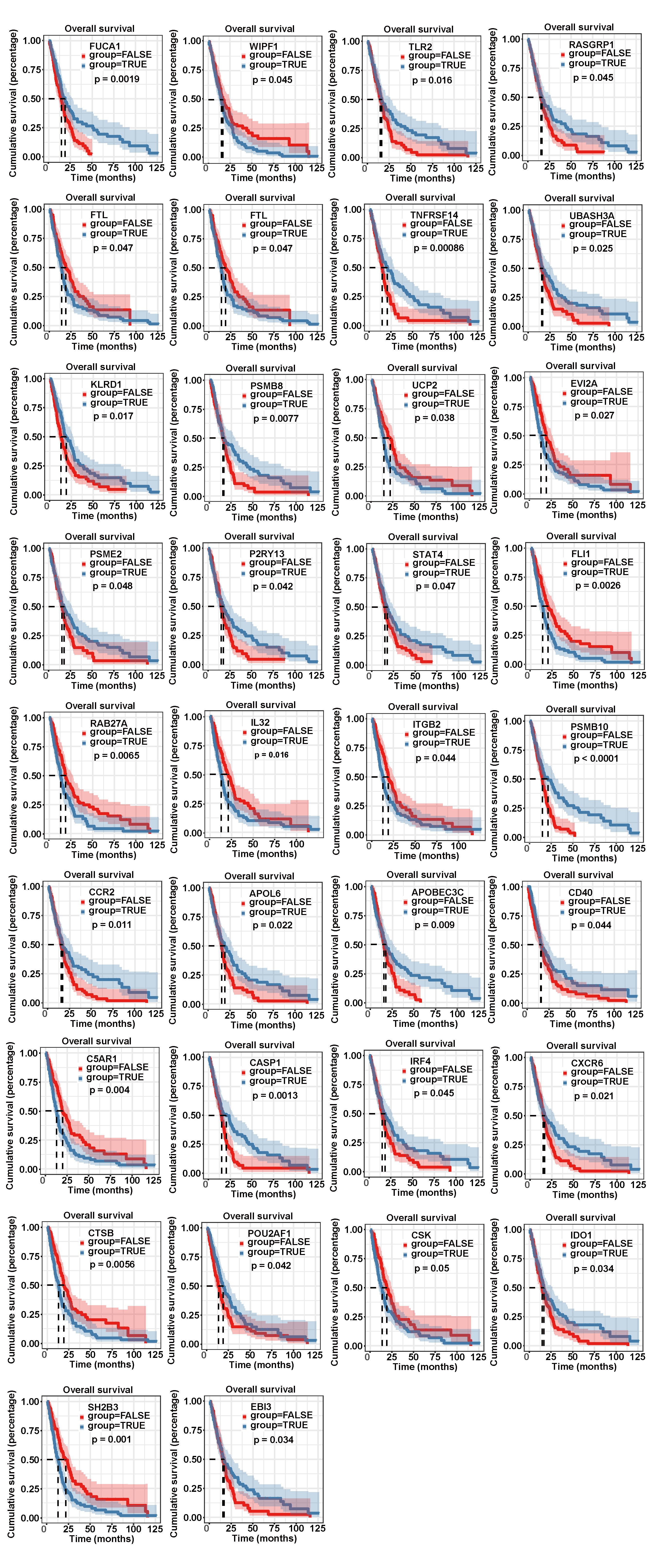


**Supplementary Figure 2:** Plotting K–M survival curves to find prognostically significant genes in the MEturquoise module.


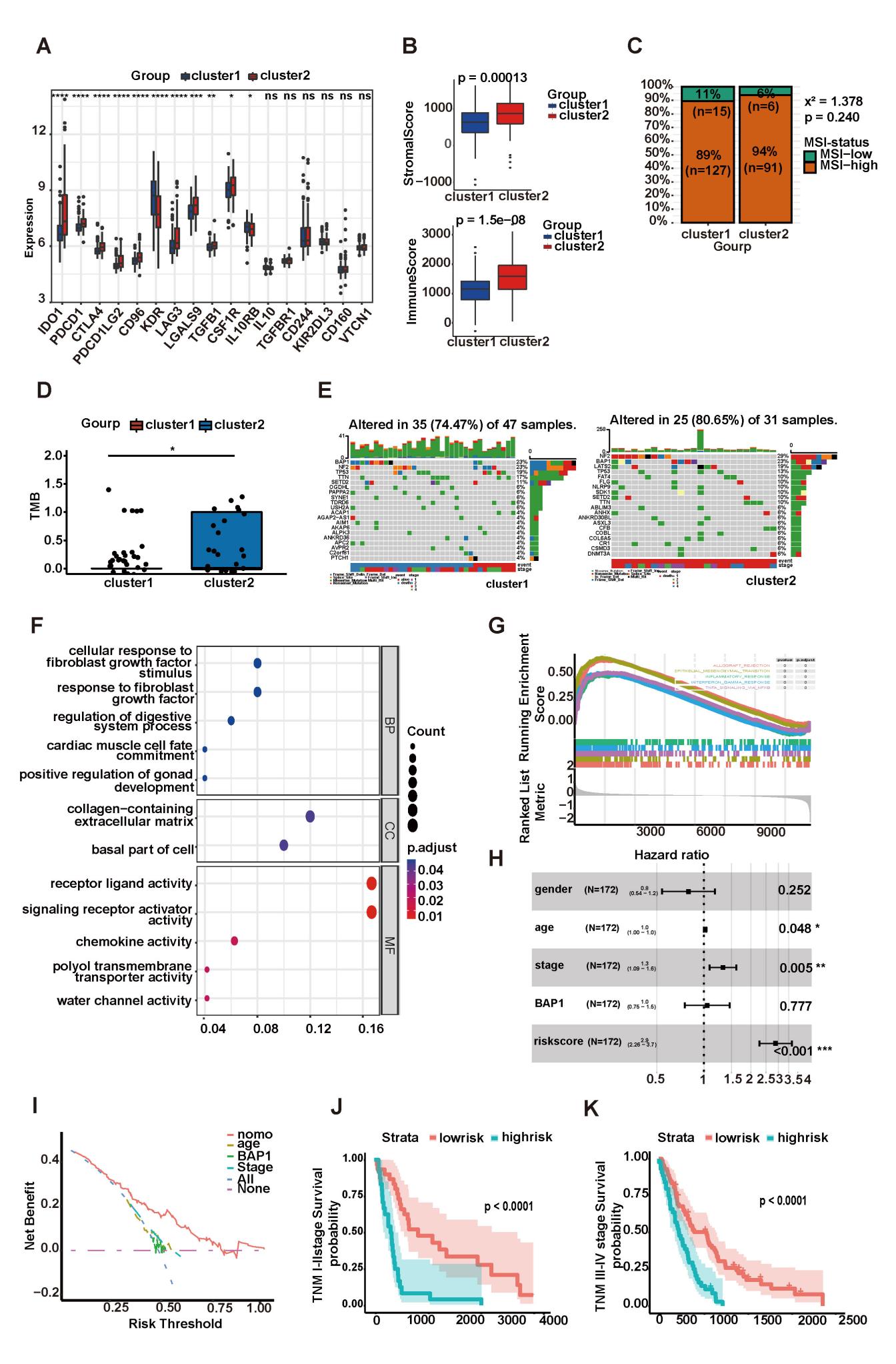


**Supplementary Figure 3:** In-depth analysis of cluster1 and cluster2 immune profiles and construction of risk score models. **(A)** Comparison of infiltration levels of immunosuppressive factors in cluster1 and cluster2. **(B)** Boxplots of immune microenvironment in cluster1 and cluster2. **(C)** Histogram of MSI in cluster1 and cluster2. **(D)** Histogram of TMB in cluster1 and cluster2. **(E)** Waterfall map of somatic mutations in cluster1 and cluster2. **(F)** GO enrichment analysis of DEGs. **(G)** Significant enrichment of EMT signals in cluster2. **(H)** Forest plot showing risk score as an independent prognostic biomarker by using multivariate analysis. **(I)** DCA charts for assessing the clinical utility of risk score models. **(J, K)** Risk score model prediction of OS in patients with MPM with different TNM stages. (* *p* < 0.05, ** *p* < 0.01, *** *p* < 0.001)


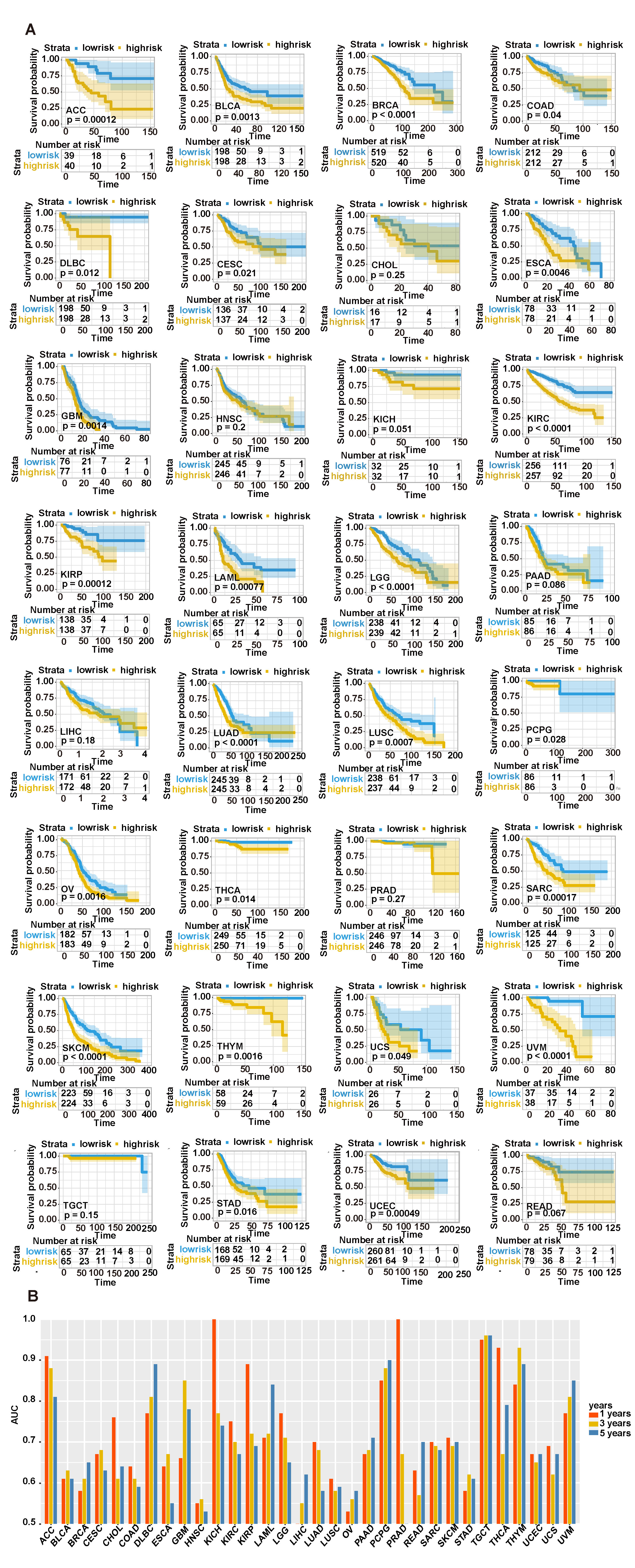


**Supplementary Figure 4:** Predictive power of risk score models at pan-cancer level.**(A)** Survival analysis of patients in low-risk and high-risk groups of TCGA pan-cancer cohort. **(B)** Predictive performance of ROC curve assessment risk score models in pan-cancer.


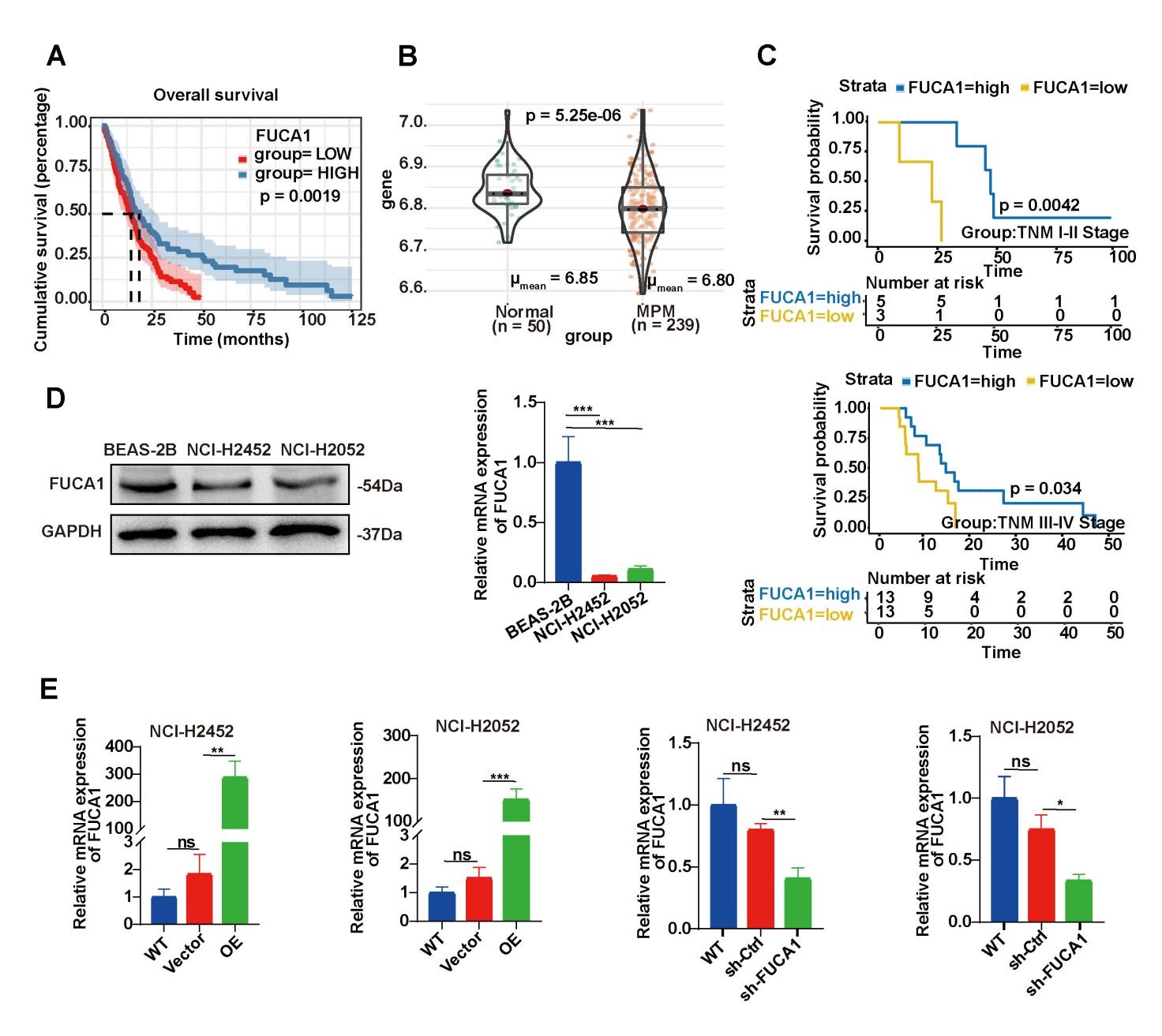


**Supplementary Figure 5:** Expression of FUCA1 in MPM tissues and cells.**(A)** Survival analysis of different FUCA1 expression groups in patients with MPM in TCGA and GEO databases. **(B)** Expression of FUCA1 in MPM cancer tissues and matched paracancerous tissues. **(C)** Relationship between FUCA1 expression and OS in different TNM subgroups. **(D)** Expression of FUCA1 in BEAS-2B, NCI-H2452, and NCI-H2052 cell lines. **(E)** qRT-PCR validation of FUCA1 transfection efficiency.


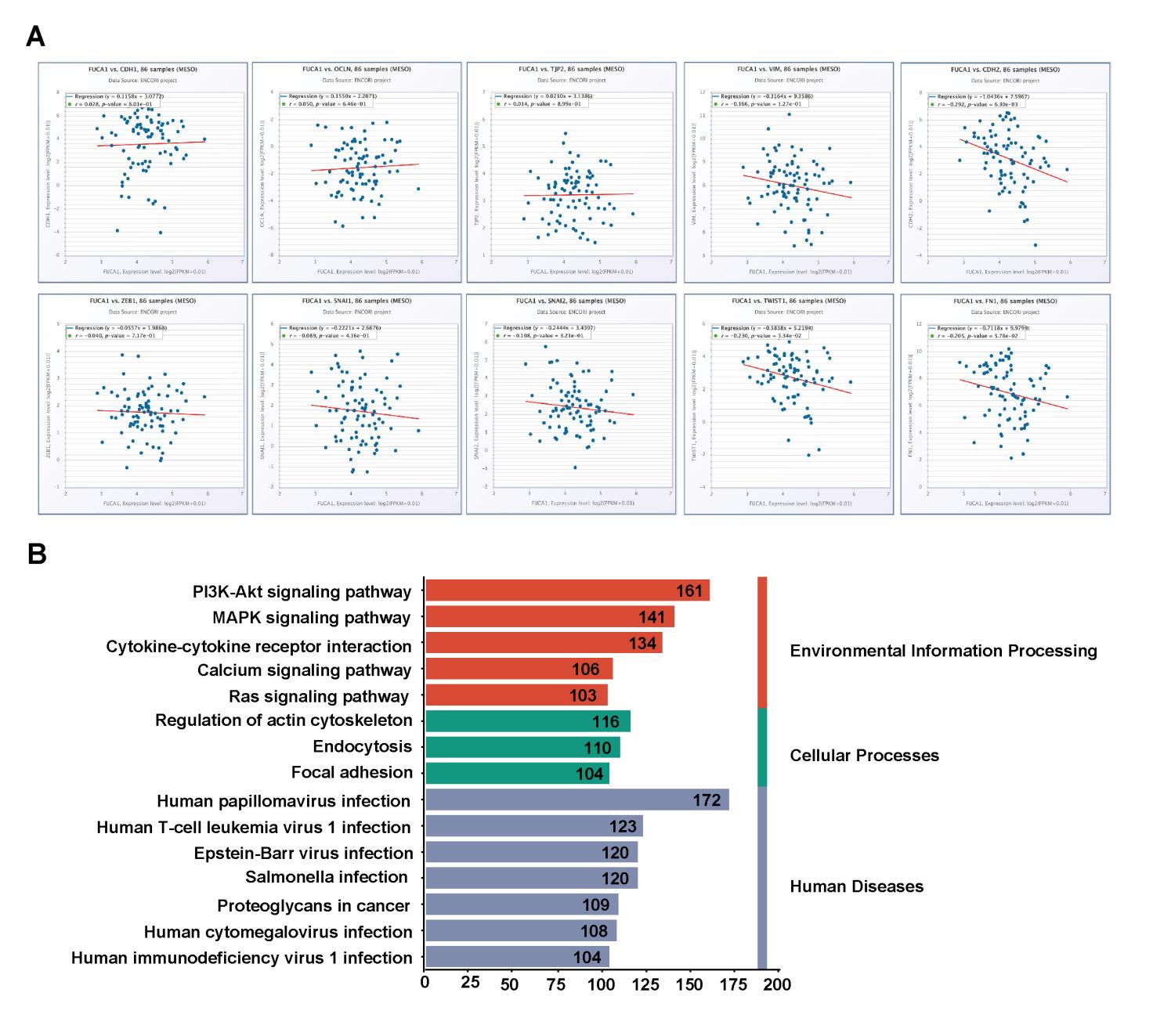


**Supplementary Figure 6:** Bioinformatics analysis indicating that FUCA1 is associated with EMT and PI3K-AKT signaling pathways.**(A)** Correlation analysis of FUCA1 expression with EMT in the TCGA database. **(B)** KEGG pathway enrichment analysis.





**Supplementary Figure 7:** Statistical analysis of protein expression levels. **(A)** Relative protein expression levels of Snail/GAPDH, Vimentin/GAPDH, E-Cadherin/GAPDH, and FUCA1/GAPDH. **(B)** Relative protein expression levels of AKT/GAPDH, P-AKT/GAPDH, PI3K/GAPDH, and P-PI3K/GAPDH. **(C)** Relative protein expression levels of P-AKT/GAPDH, Snail/GAPDH, Vimentin/GAPDH, and FUCA1/GAPDH. All data are presented as mean ± SD (n = 3). Statistical significance was determined using one-way ANOVA followed by Tukey's post-hoc test. (ns : no significance, * *p* < 0.05, ** *p* < 0.01, *** *p* < 0.001, **** *p* < 0.0001).
